# A review on conducting organic polymers: Concepts, applications, and potential environmental benefits

**DOI:** 10.1016/j.heliyon.2025.e42375

**Published:** 2025-01-30

**Authors:** Md. Byzed Hasan, Md. Masud Parvez, Abrar Yasir Abir, Md. Faruak Ahmad

**Affiliations:** a Department of Chemistry, Pabna University of Science and Technology, Pabna-6600, Bangladesh; b Department of Chemistry, University of Barishal, Barishal-8254, Bangladesh

**Keywords:** Conducting polymer, Doping, Conduction mechanism, Solar cell, Fuel cell, Supercapacitor, Led, Heavy metal, Wastewater, Adsorption, Photocatalysis

## Abstract

Polymer materials have long been valued for their insulating properties. But recent advancements have revealed their potential as electrically conductive materials, offering an alternative to traditional metallic conductors with the added benefit of reduced environmental impact. This review article provides a comprehensive overview of conducting organic polymers, focusing on their conceptual foundations, diverse applications, and their significant role in mitigating environmental pollution. The paper begins with an exploration of how polymeric materials have progressed from insulators to conductors, explaining the basic principles and mechanisms behind their electrical conductivity. It then provides an insight into the various applications enabled by their unique optical and electronic properties, including their use in light-emitting diodes, electrochromic displays, smart windows, fuel cells, solar cells, supercapacitors and batteries. Additionally, the review emphasizes the potential of conducting organic polymers in mitigating environmental pollution, particularly through their role in wastewater treatment and e-waste management. By examining recent advancements and promising future prospects, this article underscores the potential of conducting organic polymers to revolutionize both electronic technology and environmental sustainability.

## Highlights

• A comprehensive exploration of conducting organic polymers.
• Describes the fundamental mechanism that gives rise to their electrical conductivity.
• Outlines the role of their optical properties in LEDs and photovoltaic cells.
• Draws attention to the recent researches being done on their possible uses.
• Insight into the mechanisms by which they contribute to waste water treatment.

## Graphical abstract

**Figure:**
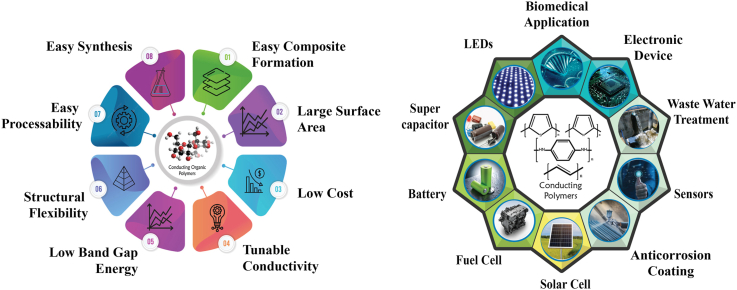

## 1 Introduction

Conducting polymers (CPs), sometimes referred to as conductive organic polymers, are a type of organic polymeric materials that exhibit electrical and optical characteristics similar to those of inorganic semiconductors and metals. CPs have garnered a lot of interest due to their economic importance, outstanding environmental stability, electrical conductivity, necessary mechanical, optical, and electronic properties, as well as their eco-friendly nature [1,2].

The insulating capabilities of polymeric materials have long been recognized. Conductivities of 10^−20^ S/m are achieved by polytetrafluoroethylene with the repeating unit -CF_2_CF_2_- or poly (vinyl chloride) with the repeating unit -CH_2_CHCl-. Copper has a conductivity of around 5.96 × 10^7^ S/m on the other end of the scale [3]. Recent research over the past few decades has demonstrated that conjugated polymers can be engineered to achieve conductivities comparable to those of highly conductive metals. In 1862, Henry Letheby, a professor of Chemistry at the College of the London Hospital, created partially conducting polyaniline by anodic oxidation of a sulphatic aniline solution [4]. Subsequently, in 1977, Alan J. Heeger, Alan MacDiarmid, and Hideki Shirakawa reported the synthesis of semiconducting polyacetylene. When this semiconducting polyacetylene is exposed to halogen vapor (Cl_2_, Br_2_ or I_2_), uptake of halogen occurs, and the conductivity increases markedly. In the case of I_2_ vapor, the conductivity of polyacetylene is found to be nearly of the same order as metal [5]. Since then, research on conducting organic polymers has been increasing gradually and the discoverers of electrically conducting polyacetylene were awarded the 2000 Nobel Prize in Chemistry for the “discovery and development of electrically conductive polymers.”

Electronic wastes, also known as e-waste, waste electrical and electronic equipment (WEEE), or end-of-life (EOL) electronics, are the waste produced by obsolete/discarded electrical appliances or electronic products such as refrigerators, washing machines, fans, lights, televisions, air conditioners, batteries, cell phones, and computers [[6], [7], [8]]. Electrical and electronic equipment is one of the global industries with the quickest rate of growth due to the rise of globalization, industrialization, economic prosperity, technical advancement, and affluent lifestyles. The amount of WEEE has increased as a result of this progression. E-waste releases hazardous heavy metals (Pb, Sb, Ba, Cd, Ni, Hg, Cr, and As) into the environment. Because of their toxicity, bioaccumulative nature, and endurance in the environment, the heavy metals identified in e-waste are pose a serious threat to our environment. Some metals and their ions interfere with biological functions and growth, while others accumulate in one or more organs, causing a a number serious diseases including respiratory tract irritation, kidney and liver damage, weakened immune systems, and nasal, sinus or lung cancer caused by chromium (Cr); nervous cell apoptosis, memory loss, immune toxicity, and muscle weakness caused by mercury (Hg); dermatitis and bronchial asthma caused by nickel (Ni); anemia, nephrotoxicity, nervous system damage, and hypertension caused by lead (Pb); and kidney injury, decreased bone density, lung damage, cancer caused by cadmium (Cd) [[9], [10], [11]]. Globally, the rate of e-waste generation has increased by over 2 metric tonnes (Mt) every year. By 2030, 74 Mt of e-waste is expected to be generated [12]. The use of conducting polymers instead of metal conductors will go a long way in reducing heavy metal pollution in the environment.

Organic dyes, petroleum derivatives, pesticides, and toxic medications can all be found along with heavy metals and their ions in the wastewater produced by a variety of industries, including paint and paper, clothing and textile, metallurgy, petrochemical, fertilizer, and semiconductor manufacturing [[13], [14], [15], [16], [17], [18]]. Wastewater containing such carcinogenic and toxic organic pollutants must be treated before releasing it into the environment. Conducting organic polymers and their composites have a great deal of success in removing harmful metal ions and organic pollutants from wastewater by adsorption and photocatalytic degradation, in addition to carbon nanotubes, plant-based adsorbents, semiconducting metal oxides, graphitic carbon nitride (g-C_3_N_4_), and polymer hydrogel-based nanocomposites, which are all well-established materials to treat wastewater [[19], [20], [21], [22], [23]].

Furthermore, due to their low density, flexibility, low band gap energy, ease of synthesis, ease of processing, tunable conductivity, and environmental friendliness, CPs are promising candidates for a variety of applications. As a result, during the past several decades, researchers have concentrated more on CPs and the many composites they form in an attempt to find unique properties that aren't present in individual materials. To better understand the distinct behavior and operation of these materials and their wide range of applications, we will effectively discuss the various properties, working principles, applications, and role of conducting polymers in reducing environmental pollution here in this thorough review. To the best of our knowledge, we are the first to write a review paper of this type that properly addresses the function of CPs in reducing environmental pollution. The insights from this paper will enable future researchers to design more effective CP-related studies by providing a clearer understanding of the characteristics, mechanisms, and uses of conducting organic polymers.

## 2 Conducting organic polymers

Conducting organic polymer can conduct electricity by means of the presence of extended and delocalized conjugation in their polymeric backbone, which arises from the overlap of π-electrons. Nowadays, they are attracting increasing interest due to their ease of processing and controllable electrical conductivity. CPs are often referred to as intrinsically conducting polymers (ICP), as the polymer materials themselves [24] conduct electricity without the incorporation of any conducting substance. The conductivity of ICPs can be controlled by adding a suitable contaminant or doping agent. Besides, there have already been some reports of extrinsically conducting polymers, which are actually composites of a conducting substance and a non-conducting polymer [25]. In this review article, we will limit our focus only to intrinsically conducting polymers.

### 2.1 Polyacetylene

The development of polyacetylene and the increase of its conductivity through doping with the appropriate doping agent earned Alan J. Heeger, Alan MacDiarmid, and Hideki Shirakawa the 2000 Noble Prize in Chemistry. In polyacetylene, the long chain of carbon atoms alternates between single and double bonds, with the double bond having a choice of cis or trans geometry [Scheme 2**]** depending on the temperature at which the polymerization reaction is carried out. Each carbon atom in the backbone of the polymer has one hydrogen, which can be replaced with the proper functional group to increase the polymer's stiffness, solubility, geometry of the double bond, and length of effective conjugation [26].

Polyacetylene, in its pure form, is not a conductor (conductivity is in the range of 10^−5^ S/cm for *trans* isomer while 10^−9^ S/cm for *cis* isomer) [27]. However, by doping polyacetylene with a very small amount of suitable electron-donating (n-doping) or electron-accepting (p-doping) agent it is possible to enhance the conductivity, and such polycatenane shows conductivity nearly of the same order (>10^5^ S/cm) of highly conductive metal like copper [5,[27], [28], [29], [30]].

### 2.2 Poly (p-phenylene vinylene)

**Scheme 1:**
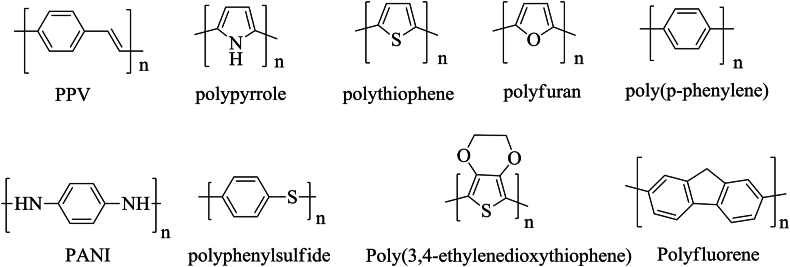
Skeleton formula of some common conductive polymers.

Poly (p-phenylene vinylene) (PPV) is a highly crystalline rigid-rod-like [31] polymer having phenyl substituted vinylene repeating unit (Scheme 1). Polyacetylene, when undoped, is an insulator with a conductivity value of only 10^−13^ Scm^−1^, but upon doping PPV shows conductivity similar to that of doped polyacetylene, and conductivity approaches to a value of 10^2^ Scm^−1^ to 10^4^ Scm^−1^ depending on stretch ratio [32] when dopped with H_2_SO_4._ Though it approaches a saturation point above a conjugation length of around six units, the conductivity of PPV grows as a function of conjugation length [33]. The high conductivity with a very weak temperature dependency indicates that dopped PPV is a metal-like electronic conductor [32].

**Scheme 2:**
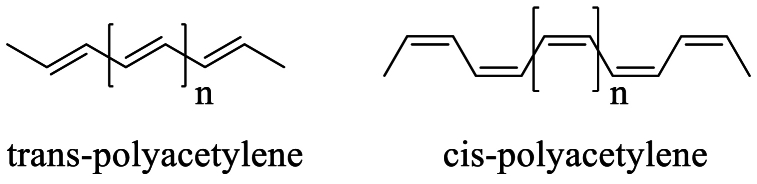
Skeleton formula of cis- and trans-polyacetylene.

### 2.3 Polyaniline

Although polyacetylene is first mentioned in the discussion of conducting polymers, polyaniline [Scheme 1] is the first conjugated organic polymer to be discovered. Prior to the discovery of its conductivity, it was investigated as a number of colorful compounds and dyes [34]. The conductivity of polyaniline was brought to light by Alan MacDiarmid in the 1980s. He along with his coworkers synthesized polyaniline (PANI) from an aqueous solution of aniline via both chemical and electrochemical processes, which in its oxidized form upon doping with a protonic acid (p-dopped) found to exhibit electrical conductivity [35].

**Scheme 3:**
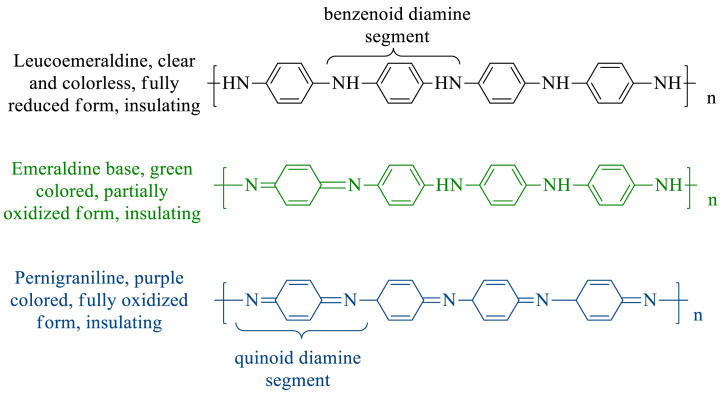
Structural illustration of various forms of PANI.

PANI exists in three different forms with varying oxidation states [36] and colors, i.e., 1. Leucoemeraldine, 2. Emeraldine, and 3. Pernigraniline [Scheme 3]. PANI's oxidation state is recognized to have a significant impact on its chemical, structural, electrical, and optical characteristics [37]. The ratio of benzenoid diamine part (reduced) and quinoid diamine (oxidized) part is 0.5 in emeraldine, whereas benzenoid diamine and quinoid diamine part is the only repeating unit in leucoemeraldine and pergraniline respectively. They are sometimes referred to as leucoemeraldine base (LB), emeraldine base (EB), and pernigraniline base (PB), as they can be protonated when dopped with a Brønsted acid. PANI has the capacity to store chemical energy in the form of electrical energy during these redox transitions between the bases of leucoemeraldine, emeraldine, and pernigraniline [38,39].

**Scheme 4:**
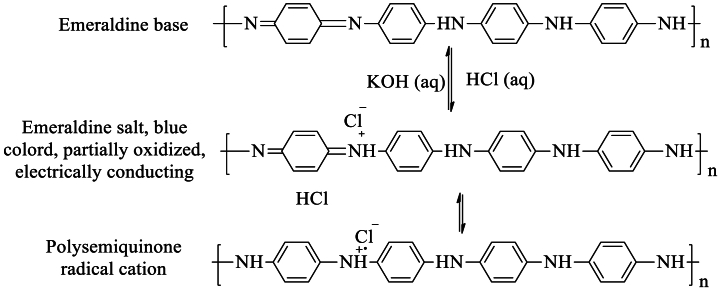
Formation of Emeraldine salt from Emeraldine base.

PANI becomes conductive when dopped in its partially oxidized forms (emeraldine base) only [40]. Even after being doped with protonic acid, the other two forms, LB and PB, are poor conductors of electricity. Emeraldine base is the most studied form of polyaniline among its three main forms because of its excellent stability at room temperature. When doped with an acid (p-doped), it transforms into the emeraldine salt (ES) form [Scheme 4**]**, which exhibits strong electrical conductivity due to the protonation of the imine nitrogen [41]. The non-conjugated emeraldine salts become conjugated after doping and create charge carriers in the form of polysemiquinone radical cation, which is why ES becomes conductive after doping with an increase of conductivity by 10^10^ times [[42], [43], [44]]. Therefore, polyaniline is different from other CPs in that way that like other polymers PANI doesn't need to be doped by a doping agent that can add electrons to or remove electrons from the polymeric material.

Furthermore, unlike all other conducting polymers, the conductivity of ES relies not only on the degree of oxidation of the polyaniline but also on the degree of protonation of the material. The conductivity of ES rises as the pH of the solution used to protonate the EB polymer decreases, eventually reaching a saturation value at a pH below 0 [43].

Due to its mechanical qualities, affordable production costs, durability of conducting forms in the presence of air and moisture [35], high thermal stability [45], and distinctive conductive characteristics, PANI is gaining interest compared to other conjugated polymers.

### 2.4 Polypyrrole

Polpyrrole (PPy) **[**Scheme 1], an organic polymer made from pyrrole by chemical [46] and electrochemical [47] synthesis, is used in the domains of electronics, optics, biology, and medicine. Indeed, even though polyaniline was the first organic polymer ever synthesized, PPy is the first reported organic conducting polymer [48,49]. In 1963, DE Weiss and his coworkers found that iodine-doped PPy exhibits good electrical conductivity [[50], [51], [52]].

In the late 1970s, pyrrole was electrochemically polymerized on a platinum surface in a two-electrode cell filled with 0.1 M Et_4_NBF_4_ and 0.06 M pyrrole in a 99 % aqueous solution of acetonitrile. Arthur F. Diaz and his coworkers at IBM synthesized a strongly adhered, durable, and thermally stable PPy film with a conductivity range from 10 to 100 Scm^−1^ at room temperature [53,54]. Polymer synthesized in this manner was found to carry a positive charge (hole). Electrochemically polymerized PPy films were also found to follow a temperature profile consistent with a traditional p-type semiconductor, i.e., conductivity increases with temperature [[54], [55], [56]]. The conductivity of PPy film depends on some factors: polymerization temperature, polymerization technique, additives used during polymerization, film stretching, counterions present in the polymer backbone, oxidant to pyrrole ratio, reaction temperature, and reaction time [[57], [58], [59], [60]].

### 2.5 Polythiophene

Polythiophene (PTh) is a highly conductive organic polymer comprising thiophene repeating units [Scheme 1], which are connected with each other through 2- and 5-position. Because of its excellent electronic and optical properties, the application of polythiophenes in electrical devices such as light-emitting diodes (LEDs), water purification systems, hydrogen storage, and biosensors is a topic of extensive research [[61], [62], [63], [64]]. Polythiophene, in its pure form, is a poor conductor of electricity like most other organic polymers. However, doping with a proper doping agent can markedly enhance its conductivity, though *n*-doped PThs are rarely observed [65]. The *p*-type doping agent creates a positively charged unit called bipolaron through which it is possible to produce a highly conductive polymer. FeCl_4_^−^ dopped PTh films have a maximum conductivity of 50 Scm^−1^ [66], and a conductivity of 100 Scm^−1^ can be achieved when dopped with BF_4_^−^ [67]. Conductivity for Poly (3-dodecylthiophene), a substituted polythiophene, is found to approach a value of approximately 1000 Scm^−1^ [68], which is quite enough for many applications.

3,4-ethylenedioxythiophene (EDOT), a substituted thiophene, upon oxidative chemical polymerization or electrochemical polymerization produces Poly (3,4-ethylenedioxythiophene) (PEDOT) [Scheme 1], a substituted PTh which also shows high electrical conductivity (up to 5400 Scm^−1^) [69] along with high stability [70,71]. Polyisothianaphthene also shows better electrical conductivity than PTh [72].

### 2.6 Poly (p-phenylene)

Poly (p-phenylene) (PPP) is an organic polymer that draws the attention of researchers due to its rigid rod-like backbone, thermal stability, and tunable electrical and optical properties. PPP consists of a linear sequence of π-conjugated *p*-phenylene repeating units [Scheme 1], which accounts for its electrical conductivity. It was once challenging to synthesize unsubstituted high molecular-weight PPP because of the difficulties of C-C bond synthesis. However, excess polycondensation in the presence of a Pd-based catalyst (Suzuki Coupling) made it easier nowadays [[73], [74], [75]]. PPP can also be synthesized using several methods, including direct electrochemical oxidation of benzene [76,77], Wurtz reaction, and Ullmann reaction. Additionally, PPP can be prepared by directly oxidizing benzene in the presence of an oxidant and a Lewis base catalyst [78].

PPP, in its poor state, is a poor conductor of electricity with a conductivity value less than 10^−14^ Scm^−1^, but doping with a suitable doping agent can markedly increase the conductivity of PPP. FeCl_3_-doped PPP shows both electronic and ionic conductivity in the range of 10^−5^ to 10^−8^ Scm^−1^ [79]. AsF_5_-doped (*p*-type doping agent) PPP was found to exhibit an electrical conductivity value of ∼10^2^ Scm^−1^, while a conductivity value of ∼10 Scm^−1^ was found when dopped with potassium (*n*-type doping agent) [80,81].

### 2.7 Polyphenylene sulfide

Polyphenylene sulfide (PPS), having phenylene repeating units connected with each other by sulfide linkages, is a semi-crystalline thermoplastic with excellent mechanical properties **[**Scheme 1**]**. PPS shows conductivity up to 3 Scm^−1^ when dopped with strong electron acceptors such as AsF_5_ and SbF_5_ [82]. The conductivity of hydrated poly (phenylene sulfide sulfonic acid) reaches 100 Scm^−1^ which drops rapidly above 100 °C due to the loss of water via evaporation [83].

### 2.8 Polyfluorene (PF)

With applications in LEDs, lasers, solar cells, memory, field-effect transistors, and sensors, polyfluorene-composed of fluorene repeating units [Scheme 1**]**- has become a diverse class of semiconducting materials [84]. Undoped PF, synthesized by the electrochemical oxidation polymerization of fluorene, has a conductivity value of 10^−4^ Scm^−1^ [85], which can be increased markedly upon doping with a suitable dopant.

The electroluminescence property of PF, which results from enhanced π-conjugation [86], as well as the suitable band gap energy of PF that ranges from 2.8 eV to 3.5 eV [87] makes PF an excellent candidate for making electronic devices such as organic solar cells [88], organic light emitting diodes [89], organic thin film transistors [90], and biosensors [91].

### 2.9 Superconducting polymer

**Scheme 5:**
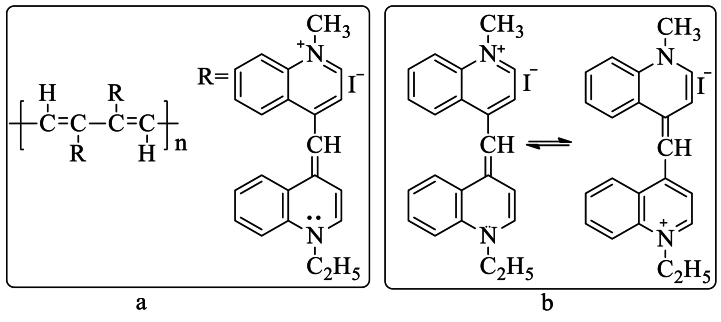
a. Chemical structure of the proposed super conducting organic polymer. b. Resonance structure of the side chain [93].

A superconductor is a substance with a specific critical temperature (Tc) below which resistance quickly decreases to zero and conduction reverts to those of an ordinary metal above Tc. An electric current in a superconducting loop can last eternally without being powered [92]. In 1964, W.A. Little [93] suggested that superconductivity in organic polymer can be achieved even at temperatures well above room temperature. With his theoretical calculation, he showed, using a specific polymeric model having a polyene system as a polymeric backbone containing a very well-known dye molecule named diethyl cyanine iodide as a side group [Scheme 5**],** that the critical temperature could be several hundred kelvins for such a polymer.

Unfortunately, there have been no practical reports of organic polymers demonstrating superconductivity to date. However, the first reported polymer to exhibit superconductivity is poly (sulfur nitride), also known as polythiazyl, (SN)_x_, though an inorganic polymer, which was found to lose electrical resistivity below 0.26 K [94]. Therefore, the first superconducting polymer was found to have exhibited superconductivity very far from room temperature.

## 3 Mechanism of electrical conductivity

### 3.1 Conduction by inherent conjugation and doping

**Fig. 1:**
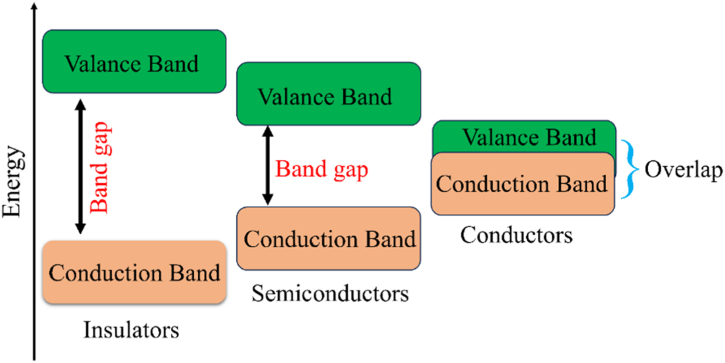
Electronic band structures of metals, semiconductors, and insulators.

Band theory explains the electrical characteristics of any substance. Molecular orbital theory is the foundation of this theory. This theory states that a material's valence orbitals interact with one another to produce a set of molecular orbitals known as bands that extend throughout the entire substance. Similar to the valence electrons of individual atoms, the band of the highest occupied molecular orbital (HUMO) of a material is termed the valence band (VB) [Fig. 1**]**. Since current in these materials can only flow when electrons are stimulated to this band, the band of lowest unoccupied molecular orbital (LUMO) is known as the conduction band (CB). The forbidden band, often referred to as the forbidden gap, is the energy gap that exists between the valence band and the conduction band. The electrical conductivity of a solid is determined by the forbidden gap. The high electrical conductivity of metals is attributed to the constant availability of electrons in their conduction band, which is caused by the fact that metals effectively have no energy gap between their valence and conduction band as the two bands overlap. Therefore, electrons are constantly available in these two closely spaced bands. An electron cannot be promoted to the conduction band in an insulator because there is a noticeable energy difference (usually >4 eV) between the valence and conduction bands. Because of their tiny conductivity value at ambient temperature, semiconductors are often insulators in nature, with a band gap small enough (usually <3.0 eV) to allow thermal energy to push a limited number of electrons from the VB to the CB to give rise to electrical conductivity. A hole is left in the valence band when an electron moves up to the conduction band. Current conduction is caused by both holes and promoted electrons. As the temperature rises, so does the conductivity of semiconductors due to the increased number of holes and promoted electrons.

**Fig. 2:**
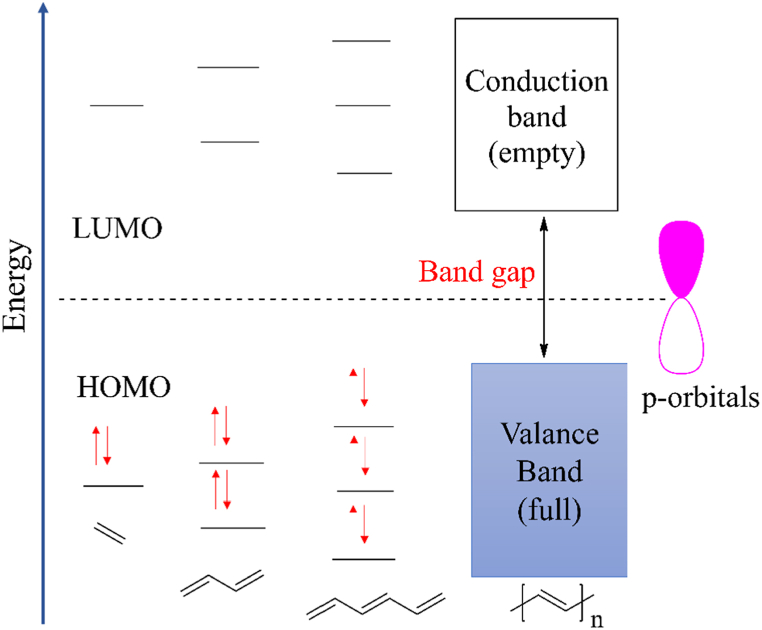
MO energy diagram for conjugated system.

CPs contain extended π-conjugation in their polymer backbone, which is why they have sufficiently smaller band gap energy (1–3 eV) [95] as the energy gap between HOMO and LUMO decreases with the increase of extended conjugation **[**Fig. 2]. For example, band gap energy for PPy at its undoped state is 3.2eV [96]. Therefore, at room temperature, thermal excitation leads to the promotion of electrons from HOMO to LUMO, which is why most undoped CPs have electrical conductivity similar to that of semiconductors.

**Fig. 3:**
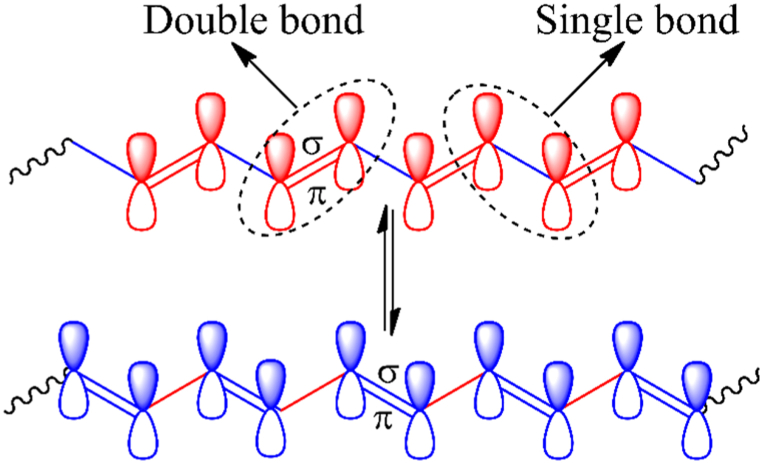
π-electron delocalization in polyacetylene.

However, the band theory is unable to provide a clear explanation for the electrical conductivity of CP [96]. Conjugated π-bonds, found in the polymer backbone of CPs, are what give them their electrical conductivity. The CPs' polymeric backbone alternates between single and double bonds. Both single and double bonds have a localized σ-bond; moreover, the double bond has a localized π-bond on top of the σ-bond. Therefore, when an electrical current is applied this localized π-electron starts moving over the polymeric backbone via delocalization **[**Fig. 3] i.e., the unhybridized p-orbitals that form the localized π-bonds now overlap continuously with the unhybridized p-orbitals of its adjacent carbon atoms, allowing current flow [95].

However, the high electrical conductivity of CPs cannot be achieved only via π-electron delocalization. High electrical conductivity requires doping CPs with the appropriate doping agent. For instance, compared to its undoped equivalent, doped polyacetylene is found to be 10^10^ times more conductive [5,[27], [28], [29]].

The standard doping notion in semiconductor physics is different from the doping concept in the case of CPs in that high conductivity results from a redox reaction in the polymeric backbone. The counterion, called dopant or doping agent, supplied by the oxidizing or reducing agent balances the charge (often called polaron or bipolaron) that the redox reaction brings into the backbone. A bipolaron is a pair of similar charges (spin 0), while a polaron is a radical ion (spin $\frac{1}{2}$) in chemical terminology [96]. Conducting polymers are therefore most often salt-like in their conductive forms. A third other charge carrier called soliton (spin can be either 0 or $\frac{1}{2}$) may also be formed. When a molecular system is ionized, its geometry changes. It is said to be degenerate if both geometries have the same energy in the ground state and non-degenerate if they have different energies. While most CPs, including PANI, PPy, and PTh, have nondegenerate ground states, trans-polyacetylene possesses a degenerate ground state [96]. When a polymer with a benzene ring in its backbone has two distinct geometries in its ground state-a benzenoid and a quinoidand the energy of the benzenoid is less than that of the quinoid, non-degeneracy results [95]. In degenerate CPs, soliton charge carriers are known to exist. On the other hand, in both non-degenerate and degenerate systems, polarons and bipolarons function as the charge carriers [95]. Electrical conductivity is facilitated by this charge carriers’ continued delocalization over the polymer backbone [96,97].

**Scheme 6:**
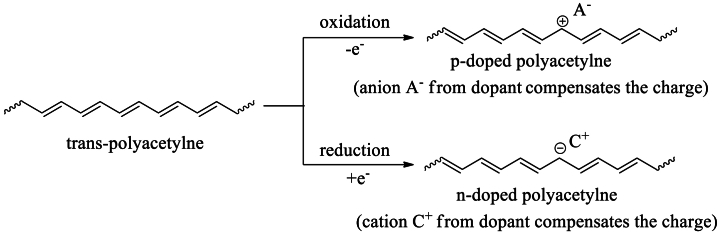
Doped *trans*-polyacetylene chain.

Both *p*- and *n*-doped CPs are possible [Scheme 6**]**, although *p*-doped CPs are more prevalent due to their greater stability over *n*-doped CP [98]. *P*-type doping eliminates one electron (oxidation) from the polymer's HOMO and increases the hole carrier density in the matrix, whereas *n*-type doping adds electrons (reduction) to the polymer's LUMO, increasing its electron carrier density. Applying an electric field increases these free-charge carriers. Consequently, doping allows for the tuning of charge carrier density and mobility [[99], [100], [101], [102]].

**Fig. 4:**
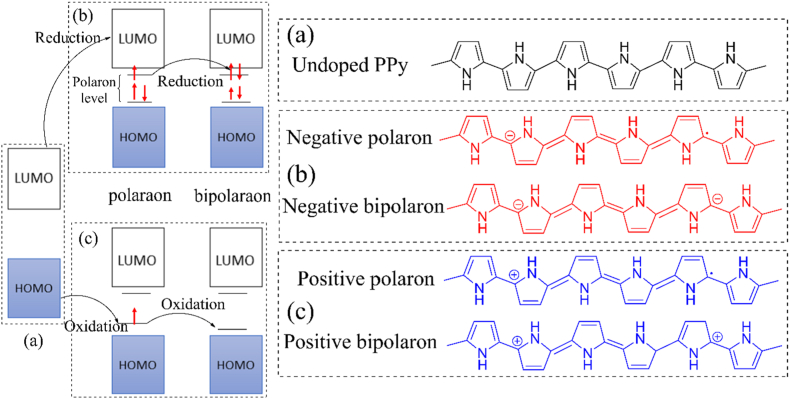
Molecular orbital and chemical structure of (a) undoped, (b) *n*-doped and (c) *p*-doped PPy [https://doi.org/10.3390/polym9040150, Open source MDPI] [95].

**Fig. 5:**
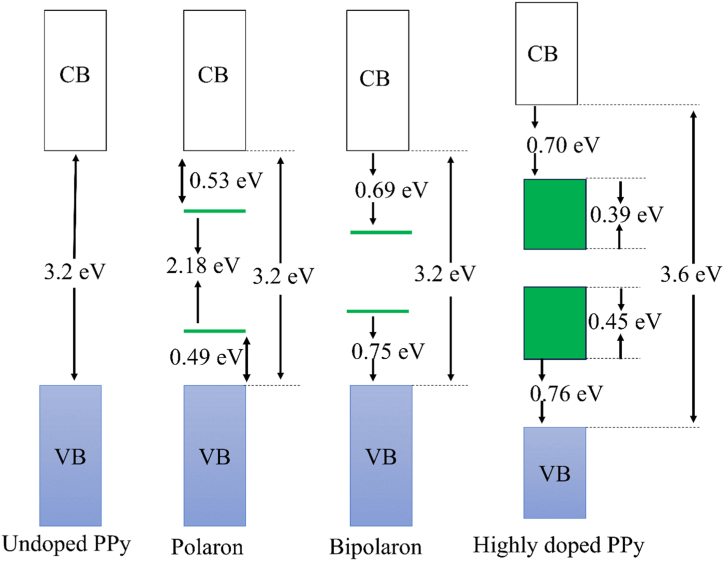
Electronic band structure of undoped and doped PPy [Adapted and modified with permission from Bredas et al. [96] Copyright © 1985, American Chemical Society.

Undoped PPy [Fig. 4(a)] is a semiconductor with a band gap energy of 3.2eV [96]. When an electron is removed from the PPy chain during oxidation (*p*-doping), a positive polaron is formed, leading to a structural deformation from a benzenoid to a quinoid structure. Therefore, two localized electronic levels, referred to as polaron levels, are created within the band gap of CB and VB. The unpaired electron so formed occupies the bonding state. Positive bipolaron is formed as a result of further oxidation [Fig. 4(c)], strengthening the benzenoid-to-quinoid deformation [95]. Also, spinless bipolaron formation is energetically favored over two separate polarons (spin $\frac{1}{2}$ for each) in the same polymer chain. Similarly, the polymer chain can be reduced to generate negative polarons and bipolarons, though n-doped polymer can't gather interest because of its low stability [Fig. 4(b)]. For p-doped PPy, the polaron levels lie about 0.5 eV and 0.75 eV away from the band edges in the case of polaron and bipolaron respectively. Further oxidation causes an overlap between the polaron levels which gives rise to the formation of two bipolaron bands of about 0.4 eV width. As the bipolaron bands formed in the band gap of CB and VB coming from the edges of CV and VB, the original band gap widens from 3.2 eV to 3.6 eV when PPy is dopped with perchlorate ion to an extent of 33 %. Although the original band gap increases, the formation of bipolaron bands lowers the energy needed to excite electrons from 3.2 eV to 1.4 eV **[**Fig. 5]. This reduction in excitation energy promotes electronic transitions and consequently improves conductivity [96].

The energy gained by the interaction with the lattice during the formation of bipolaron is higher than the Coulomb repulsion between the same charges, thereby giving it stability [96,103]. The electrical conductivity is thus facilitated by the bipolaron [96], which is analogous to the Cooper pair in BCS theory [92].

**Scheme 7:**
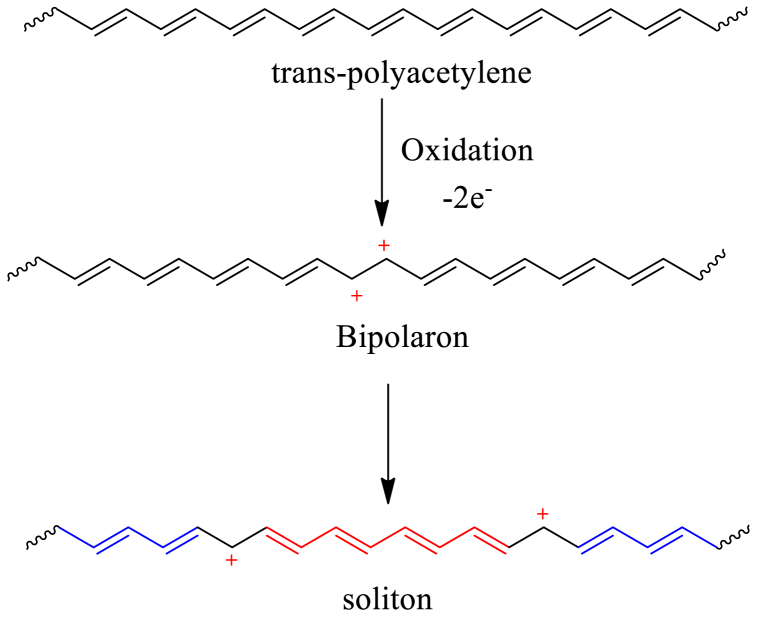
Formation of two soliton in trans-polyacetylene chain via oxidation.

**Fig. 6:**
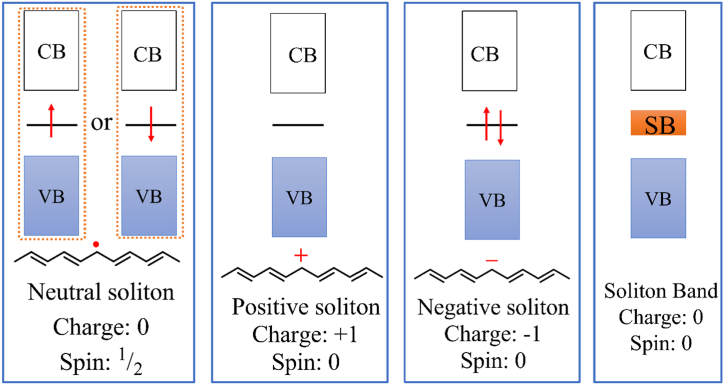
Electronic band structure of soliton in *trans*-polyacetylene.

*Trans-*polyacetylene, having a degenerate ground state unlike PPy, forms soliton instead of polaron and bipolaron upon doping. Removal of a pair of electrons by oxidation produces a bipolaron in the polymer chain which reorganizes to produce a charge-separated state, in which the two charges are separated from each other by a segment of polymeric chain [Scheme 7]. This charge separation is favored over bipolaron as the geometric structure between the two charges has the same energy as the geometry of the other sides of the charges. Because of its characteristics similar to a solitary wave that may travel without distortion or dissipation, this type of charge separation associated with a wall or barrier is known as a soliton [96]. If the pristine *trans*-polyacetylene contains an odd number of carbon atoms, π-electron delocalization from one carbon to another carbon leads to the formation of an unpaired π-electron, a radical (spin 12), which is called a neutral soliton. It is also possible for a single electron to be accepted or donated from the dopant to the polymer, resulting in the formation of a neutral soliton [104]. The neutral solitons can be destroyed by creating a double bond when they come into contact with one another as they travel along the chain. A neutral soliton can undergo oxidation (*p*-doping) to become a positive soliton, or reduction (*n*-doping) to become a negative soliton, with both states having spin 0. The soliton state forms a set of two degenerate molecular orbitals that lie in the band gap, and interaction between the charged soliton states leads to the formation of a soliton band (SB), responsible for electrical conductivity, which also located in the middle of CB and VB **[**Fig. 6]. The width of the SB increases with the increase of the doping level, and at a doping concentration above 7 % metallic-like conductivity appears because of the merging of the soliton band with the VB and CB. In addition to soliton charge carriers, electrical conductivity in *trans*-polyacetylene is also enhanced by polarons and bipolarons [95,96,104,105].

As the doping level rises, the conductivity of most of the CPs increases as well, reaching a saturated value at high doping concentrations. This is because conductivity has been found to increase with the degree of crystallinity [106], and doping causes the production of highly ordered structures [30,107].

### 3.2 Hopping conductivity and temperature dependency

**Fig. 7:**
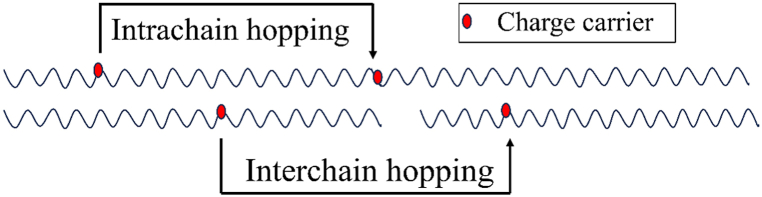
Inter and Intra chain hopping of charge carrier.

Whereas the conductivity of a conventional semiconductor falls exponentially with temperature and vanishes at absolute zero temperature, the conductivity of metal drops linearly with temperature. CPs exhibit a rise in conductivity with temperature [[108], [109], [110]] but in a different manner than that of ordinary semiconductors. The conductivity of PPy films, doped with *p*-toluenesulfonate anion, increases with temperature such that if the ln σ is plotted against T^−1/4^ it produces a straight line at high temperature [111]. The conducting mechanism in CPs is not only restricted by charge carriers’ (polaron, bipolaron, and soliton) intrachain hopping (hopping between defective areas of a single chain) but also by interchain hopping (hopping between different chains) [Fig. 7]. The intercalated ions that stabilize the charge produced by doping act as bridges that enhance the interchain hoping [112,113].

However, equation (1) for the temperature-dependent conducting behavior of CPs is derived from the Mott model of variable-range-hopping (VRH), which assumes that quantum-mechanical tunneling and phonon-assisted charge carrier hopping between localized electronic states combine to produce electronic conduction in CPs [114].

[1] $$\sigma =\sigma_{o}\;\exp\;\left[-{\left(\frac{T_{o}}{T}\right)}^{\frac{1}{d+1}}\right]$$

where σ_o_ is conductivity at room temperature, *T*_*o*_ is Mott's temperature constant, σ is conductivity at a temperature *T*, and d is the dimensionality of the system. For a three-dimensional system (d+1 = 4), equation produces:

[2] $$\ln\;\sigma \alpha \;T^{-1/4}$$

which is consistent with the conductivity of PPy films doped with *p*-toluenesulphonate anion [111]. Therefore, VRH suggests that the conductivity of CPs decreases slowly with temperature (as a function of T^−1/4^ for three-dimensional conductivity) and may not vanish at all, unlike conventional semiconductors.

However, taking conjugation length into consideration, VRH model is modified by Schaefer–Siebert and Roth [115] which yields the following equation [equation (3)]:

[3] $$\sigma =\sigma_{o}\;\exp\;\left[-{\left(\frac{T}{T_{o}}\right)}^{-\gamma }\right]$$

where the value of *T*_*o*_ and σ_o_ depend on the localization length of the wave functions and on the density off states at the Fermi level; and the value of γ, ranges from $\frac{1}{2}$ to $\frac{1}{4}$, is dependent on the dimensionality of the hopping process.

Another model, suggested by Ping Sheng [116], called the fluctuation-induced tunneling (FIT) model is also used in describing the conductivity of highly doped CPs or composites of CPs. This model assumes that if CPs contain large regions of highly conducting parts (metallic) that are separated from each other by a small region of insulating barrier (non-metallic) then electrical conduction occurs by the tunneling of charge carriers across the insulating barrier which is induced by thermally activated voltage fluctuations. The FIT model describes the temperature-dependent conducting properties by the following equations [equation (4)**]**:

[4] $$\sigma =\sigma_{o}\;\exp\;\left[-\frac{T_{1}}{{T-T}_{o}}\right]$$

where *T*_*1*_ is constant whose value depends on the properties (area, height, width and dielectric constant and effective mass of the electron) of the insulating barriers.

## 4 Optical properties

CPs have outstanding optical features, such as photoluminescence, electroluminescence, and electrochromism, in addition to their excellent electrical conducting capabilities.

**Fig. 8:**
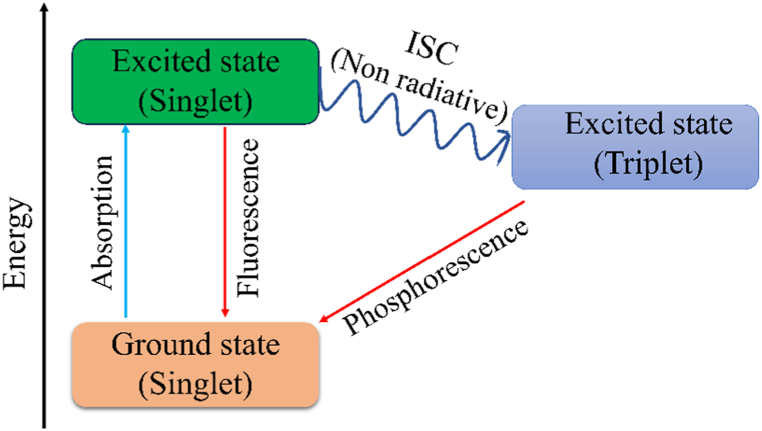
Schematic illustration of photoluminescence.

The emission of light from any substance after the absorption of photon is called photoluminescence. Because CPs have sufficiently modest band gap energies due to the existence of extended π-conjugation, electrons may be promoted from HOMO to LUMO by absorbing electromagnetic radiation of a suitable wavelength. This excited electron relaxes to the ground state by releasing excess energy in the form of photon to produce photoluminescence. Photoluminescence can be of two types: fluorescence and phosphorescence, depending on the spin of the excited electron. If the electron in the excited state has the opposite spin (singlet state) with the electron corresponding to the ground state orbital, the electron relaxes to produce fluorescence. However, the molecule may undergo an intersystem crossing (ISC) in which the excited electron changes its spin (triplet state). The emission produced when an electron in the triplet excited state relaxes back to the singlet ground state is called fluorescence [Fig. 8]. As the transition between a singlet and a triplet state is forbidden, fluorescence occurs at a slower rate compared to phosphorescence.

**Fig. 9:**
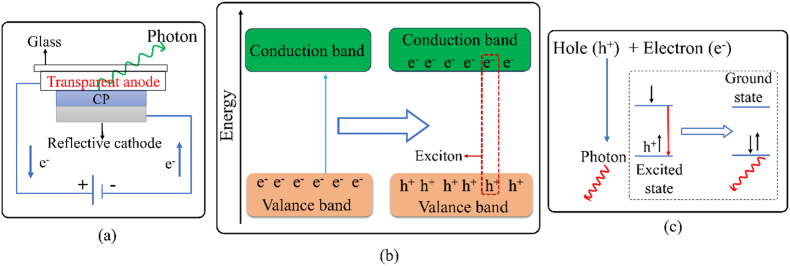
(a) Schematic representation of CP-based LED device, (b) exciton formation, (c) exciton dissociation.

Electroluminescence is an optoelectronic property of a material in which a substance emits light when an electric current is allowed to pass through it. When an electrical current is applied to a film of a CP by sandwiching it between an anode and a cathode [Fig. 9(a)], charges are introduced to the film (holes from the anode and electrons from the cathode). In order for the generated light to readily exit the light emitting diode, one electrode is often built with a reflecting metal and the other with a transparent electrically conducting substance. Usually, the cathode is built with a reflective material of low work function such as aluminum, whereas the anode is built with iridium-tin-oxide (ITO)- a transparent material with high work function. Electrical energy causes the movement of the electrons from the VB to the CB creating a hole in the valence band and an electron in the conduction band. This electron and hole which always attract each other by means of Coulombic force is often referred to as exciton (a single quasi-particle) [Fig. 9(b)], and the amount of energy that binds the electron-hole pair together is called exciton binding energy. However, the exciton-consisting of a hole and an electron-then recombines, releasing light energy [Fig. 9(c)]. The wavelength or color of the emitted light depends on the band gap energy of the CP, while the intensity of the emitted light is proportional to the amount of current flown [117,118]. Usually, the energy of the emitted photon is somewhat smaller than the band gap energy as some energy is required to break the excitonic bond. The electroluminescent property of CP is used in LED and the first reported LED based on conjugated polymer was made of poly (p-phenylene vinylene), which has a band gap energy of 2.2 eV and produces green-yellow luminescence [117]. However, the performance, brightness, color of emitted light, and lifetime of such LEDs can be improved using a combination of CPs instead of one [117,119].

Electrochromism is the ability of a material to undergo a reversible change in color and transparency when they are subjected to a small electric field (1–5 V) [120] or redox reaction [121]. Doping substances change the oxidation state of CPs, which results in the alteration of their electrochromic characteristics. PANI, reported by H. Letheby [34], was the first reported CP to exhibit electrochromic behavior as well as good electrical conductivity. PANI, in its three different oxidation states, displays three different colors [Scheme 3]. The electrochromic mechanism of CPs can be explained in terms of the doping and de-doping process. Doping agents add (n-type) or remove (p-type) electrons from the polymeric backbone of conjugated polymers, leading to changes in both their chemical structure and electronic band structure. The doping process is reversed during de-doping. Therefore, doped and de-doped (pristine) CPs show different electronic band structures which results in different colors of CPs. For instance, PANI in its partially oxidized state is green colored while it is purple colored in its fully oxidized state [Scheme 3]. When CPs are subjected to electrical potential, it causes oxidation or reduction which is analogous to doping or de-doping. The resulting charge buildup can be balanced using an electrolytic matrix. Therefore, CPs show color variation under variable electric potential. Along with PANI other CPs such as PTh, PEDOT, PPy, etc. Are found to exhibit electrochromic behavior [122,123]. This excellent optical behavior of CPs makes them attractive candidates to be used in various electrochromic devices such as multicolor electrochromic displays [124], smart windows [125], and electrochromic cells [126].

## 5 Applications of conducting polymers

### 5.1 Solar cells

**Fig. 10:**
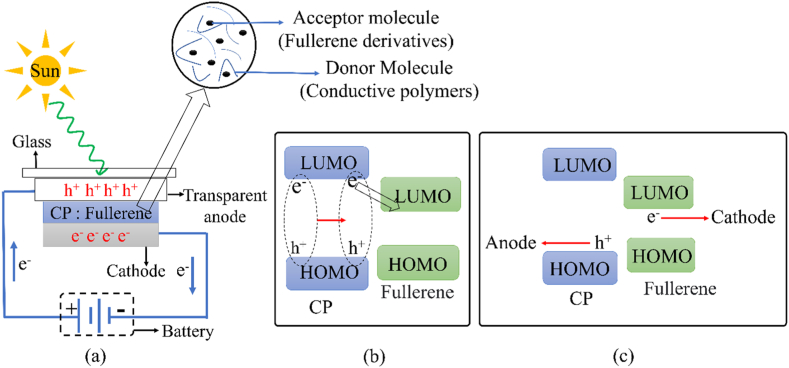
**(a)** Schematic representation of BHJ base organic solar cell, (b) Exciton formation and diffusion, and (c) Exciton dissociation and free charge carrier collection.

Solar cells are electrical devices that directly convert solar radiation into electrical energy. Nowadays, solar cells are becoming increasingly popular as a renewable and efficient energy source. Unlike fossil fuel fire stations, they don't release any greenhouse gasses or air pollution. However, because extremely poisonous nano-formed metals can be hazardous when they leak out of decommissioned solar cells and get into subterranean water supplies, their usage in modern solar cells may represent a problem [127]. CP-based solar cells are receiving a lot of interest due to their easy synthesis, lightweight, flexible structure, cheap manufacturing cost, and processability. The photovoltaic effect, which occurs when a substance is exposed to sunlight and produces an electrical current or potential between its two ends, is the basis for solar cells. The first-generation solar cell based on an organic compound was built by sandwiching a monolayer of photoconductive organic compound between two electrodes with different work functions. One of the electrodes in such a monolayer cell was transparent to allow the passage of sunlight. However, this type of monolayer cells exhibits a very low efficiency due to poor charge carrier formation and unbalanced charge transport. The initial monolayer cell exhibits efficiencies between 10^−3^ and 10^−2^ %, and exposure to 78 mWcm^−2^ of sunlight can only provide a maximum efficiency of 0.7 % when a merocyanine dye is sandwiched between aluminum and silver [[128], [129], [130]]. When sunlight hits the organic layer of a monolayer cell, it triggers an electronic transition from HOMO to LUMO, generating an exciton. To create a photocurrent, this exciton must then be separated into free charge carriers (holes and electrons). This separation can occur through various processes such as thermal excitation, autoionization, collisions between excitons, ionization by extra photons, or interactions with impurities or defects, either at the electrode-organic interface or within the bulk of the organic material [129]. The free charge carriers are subsequently collected by the electrodes, allowing electricity to flow through the external circuit. The efficiency of organic solar cells can be increased to 1 % by using a bilayer heterojunction of photoconductive organic compound instead of a single layer. Such devices can be manufactured by sandwiching two semiconducting organic materials between the electrodes [131]. In such solar cells, exciton dissociation happens at the interface of the heterojunction. Since excitons have a relatively limited lifetime, their travel distance is limited to only 5–14 nm [[132], [133], [134], [135], [136]], before decaying without producing any charge carriers, which lowers their efficiency greatly. However, the efficiency can be improved by employing the bulk heterojunction concept (BHJ). Such devices are constructed by blending a conducting polymer (donor component) with a fullerene derivative (acceptor component) [Fig. 10(a)]. Bulk heterojunction maximizes the donor-acceptor interfacial area for efficient dissociation of exciton to create a large number of charge carriers. When electrons in donor molecules are excited by light, they jump from the HOMO to the LUMO energy level. These excited electrons then transfer to the LUMO of an acceptor molecule, which is more electronegative and thus at a lower energy level, leaving behind a hole in the donor molecule's HOMO. The electron can then move between acceptor molecules, breaking up the exciton [Fig. 10(b)]. The energy difference between the donor and acceptor LUMO levels powers this electron transfer. The free charge carriers are captured by the electrodes, creating an electric current in the external circuit [137] [Fig. 10(c)]. Also, as the heterojunctions lie within a few nanometers distance from each other, the chances of exciton dissociation without creating charge carriers decrease (electron-hole recombination in a non-radiative process). The first solar cell based on BHJ was fabricated by sandwiching a thin film of polymer blend which was made by blending poly (2-methoxy-5-(2′-ethyl-hexyloxy)-1,4-phenylene vinylene) (MEH-PPV) and fullerenes [138]. Sunlight causes photoinduced generation of excitons which diffuse to reach the donor-acceptor interface. When an electron moves from the LUMO of the CP to the LUMO of the fullerene molecule, exciton dissociation takes place. The free electrons created from the dissociation of exciton then migrate to the cathode from the LUMO of the fullerene and the free holes migrate to the anode from the HOMO of the CP to create electricity in the external circuit. Usually, in a bilayer junction, the potential created at the interface of the junction due to the differences in the electron affinity of the donor and acceptor molecule provides the driving force to facilitate the dissociation of the exciton. Buckminsterfullerene C_60_ and its derivatives, such as [6,6]-phenyl-C_61_-butyric acid methyl ester PC_61_BM, have been widely employed as standard acceptors in BHJ solar cells, with conjugated polymer serving as the usual donor [139]. Because of their solubility in organic solvents and capacity for absorption in the visible region, other fullerene derivatives, such as C_70_ and its companion C_71_, have emerged as the new standard n-type materials inside active layers [140]. Such BHJ solar cells based on CP and fullerene derivatives blend exhibit a maximum power conversion efficiency of 9 % [141]. Nevertheless, by replacing fullerene derivatives with a novel class of acceptor molecules known as non-fullerene acceptors (NFAs), the power conversion efficiency can be increased by as much as 13 % [142]. The efficiency can be further improved by sandwiching a bulk heterojunction between two planar heterojunctions (PHJ), which are made by a *p*-type polymer and an *n-*type small molecule. These hybrid structures improve efficiency by obtaining enhanced charge transport and suppressing the loss of charge carriers by the recombination of holes and electrons. For instance, when a BHJ junction of PBDBTF:BTP-eC9 blend was inserted between a PHJ comprised of a p-type polymer (PTO3) and an n-type naphthalene imide molecule (NDI-i8), a maximum efficiency of 18.5 % was obtained [143]. Nevertheless, unless a significant portion of the acceptor molecule is employed to ensure a lower number of isolated acceptor molecules, the random distribution of donor and acceptor molecules over the heterojunction layer may result in electron trapping on isolated acceptor molecules resulting in the decay of charge carriers by the recombination of holes and electrons. Therefore, an ideal organic photovoltaic cell should have a well-organized donor-acceptor interface [144]. A record efficiency of 19.31 % can be achieved from a highly ordered BHJ-based solar cell whose active layer is made by blending PM6 and BTP-eC9 using 1,3,5-trichlorobenzene as a crystallization regulator [145].

### 5.2 Fuel cells

Fuel cells are considered the future energy devices that convert chemical energy stored in a fuel into electrical energy through an electrochemical reaction. Fuel cells are drawing attention because of their high energy conversion efficiency, environmental friendliness, and light weightiness.

Approximately 75 % of all normal matter is composed of hydrogen, making it the most prevalent chemical element in the universe. Hydrogen fuel cells convert the chemical energy of hydrogen directly into electrical energy, with water and heat as the resulting by-products, which do not contribute to environmental pollution. The energy density stored per unit kilogram of hydrogen is 142 MJ, which is much higher than that of any other fossil fuel or hydrocarbon fuel [146]. Therefore, Hydrogen fuel cells offer an outstanding solution for a pollution-free and highly efficient renewable energy source for the future.

**Fig. 11:**
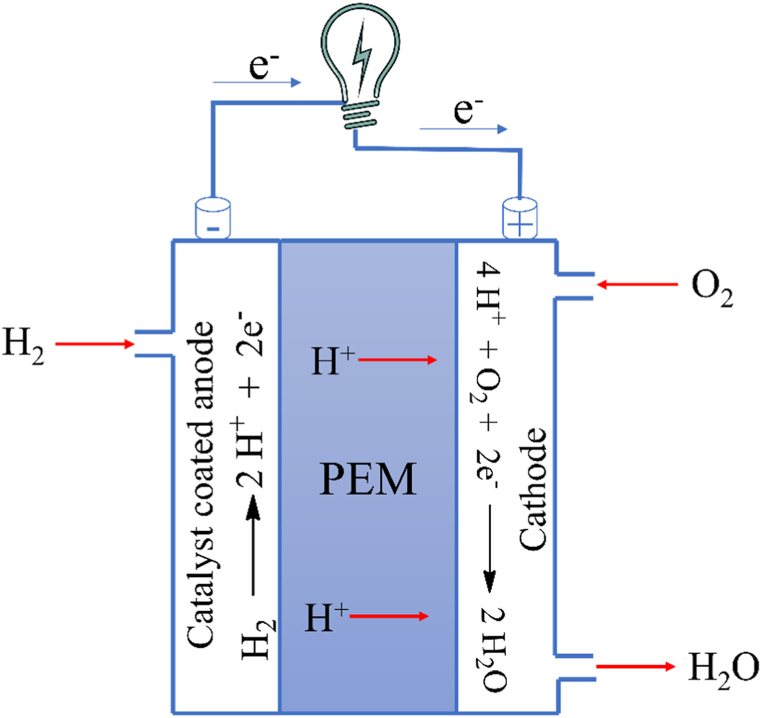
Polymer electrolyte membrane (PEM) fuel cell.

All fuel cells function similarly, although there are many different types of them. The most common type is the polymer electrolyte membrane (PEM) Fuel Cell, sometimes referred to as proton exchange membrane fuel cell (PEMFC), which has an anode, a cathode, and a membrane that conducts electrons to separate the two electrodes [Fig. 11]. A fuel cell generates electricity by utilizing the reaction between H_2_ and O_2_. Typically, protons and electrons are produced by the oxidation of hydrogen molecules when H_2_ gas is supplied to the catalyst-coated anode. These produced electrons go through the external circuit, creating a flow of electricity. The leftover protons go towards the cathode through an electrolyte, a polymer electrolyte membrane, where they mix with oxygen and electrons to form water. The electrolyte membranes have two essential functions: they “conduct” protons from the anode to the cathode and act as a barrier to prevent hydrogen and oxygen from combining directly. CPs have been thoroughly investigated as potential materials for fuel cell membranes because of their affordability, straightforward synthesis, and ability to be processed into desired morphological and microstructural forms. Additionally, they offer ease of doping and composite formation, along with chemical stability and advantageous functional properties [147].

Direct Methanol Fuel Cells (DMFCs) are a different kind of fuel cell that is becoming more and more popular these days due to the high energy density, ease of transport and storage, increased efficiency, low exhaustion, eco-friendliness, renewable nature of methanol, and ability to run at room temperature [148,149]. In DMFC, the fuel methanol is oxidized at the surface of a catalyst-supported anode in the presence of water to form protons, electrons, and carbon dioxide. The produced electron is then transported to the cathode through the external circuit where oxygen is reduced to form water in combination with the proton. The electrocatalytic anodic oxidation of methanol is one of the key elements influencing the performance of DMFC. 1D-nanostructured polyaniline and electrocatalyst based on the composite of polyaniline play a vital role in methanol electro-oxidation reaction D-nanostructured polyaniline and polyaniline-based electrocatalyst composites are crucial for the methanol electro-oxidation reaction, making them promising candidates for electrocatalyst supports in DMFCs [2,150].

### 5.3 Batteries

**Fig. 12:**
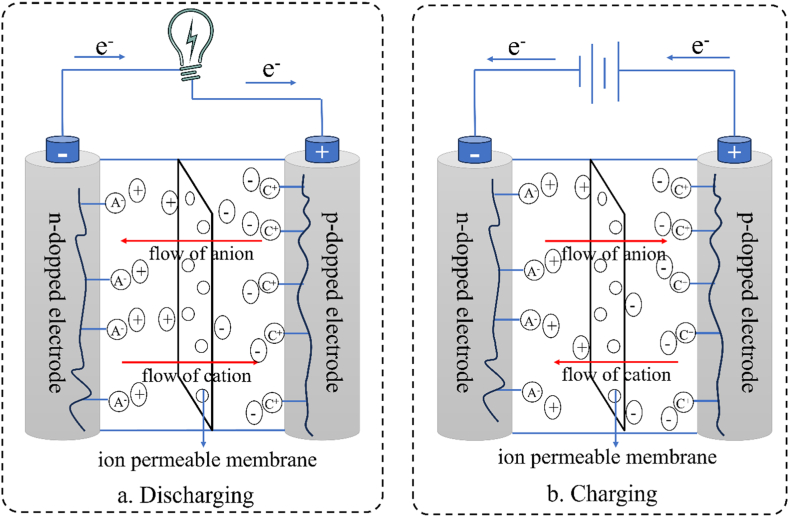
CP-based battery: a. discharging process and b. charging process.

**Fig. 13:**
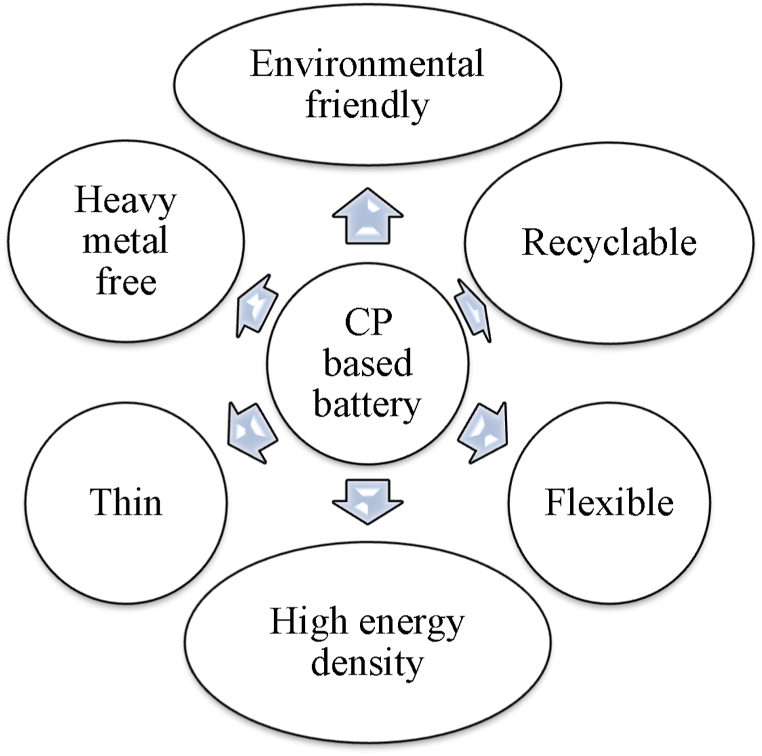
Advantages of CP based batteries.

Batteries based on CPs are advantageous over heavy metal-based batteries in terms of availability, lightweight, low thickness, low cost, and environmental considerations [Fig. 13]. Conducting polymer-based batteries use redox-active electrically conductive organic polymers as either of the electrodes or both. Usually, a p-dopped CP serves as the positive terminal while a n-dopped CP serves as the negative terminal in such a battery. The electrodes, separated by an ion-permeable separator, are dipped into an electrolytic solution which transports the ions between the electrodes [Fig. 12]. Conducting polymers contain redox-active groups, with the exception of sulfur polymers [151]. The doping process triggers redox reactions that create positive or negative ions along the polymer backbone. These ions are balanced by counterions from the dopant. P-type materials experience oxidation during charging, resulting in the formation of cations, while n-type materials undergo reduction, leading to the production of anions [Fig. 12(a)]. During discharge, the reverse processes occur [Fig. 12(b)]. To ensure charge neutrality, the electrolytic solution facilitates the transfer of counterions between electrodes through an ion-permeable separator. Remarkably, conducting polymer-based batteries with two electrodes made entirely of conducting polymers don't always require any specific types of ions unlike lithium-ion batteries, which require lithium ion. Consequently, batteries can be designed without metals and metal ions by using organic electrolytes, such as tetrabutylammonium salts, in place of conventional metal salts [152]. Nevertheless, electrodes based on CPs can also be combined with a metal-based electrode such as Zn, Li, or other metals to produce a battery.

Conventional rechargeable nickel-metal hydride or nickel-cadmium batteries have limited lifespans and capacities; in their stead, lighter Li-ion batteries with greater capacity are becoming increasingly popular [[153], [154], [155], [156], [157]]. The synergistic interaction between CPs and inorganic compounds enhances the mechanical and thermal durability of electrodes used in Li-ion batteries, as well as their lifespan, rate capabilities, and voltage. Consequently, there is a lot of interest in CPs and their composites for Li-ion batteries. Also, because of their conducting backbone, electrical conductivity, and high coulombic efficiency-which allows them to withstand hundreds to thousands of cycles without experiencing significant degradation- CPs are perfect materials for hybrid composites in Li-ion batteries [158]. Similarly, a great deal of research has been done on the synergistic interaction between nanostructured conducting polymers (NCPs) and inorganic compounds for the design of nano-batteries [159]. Fig. 13. Advantages of CP based batteries.

### 5.4 Supercapacitor

**Fig. 14:**
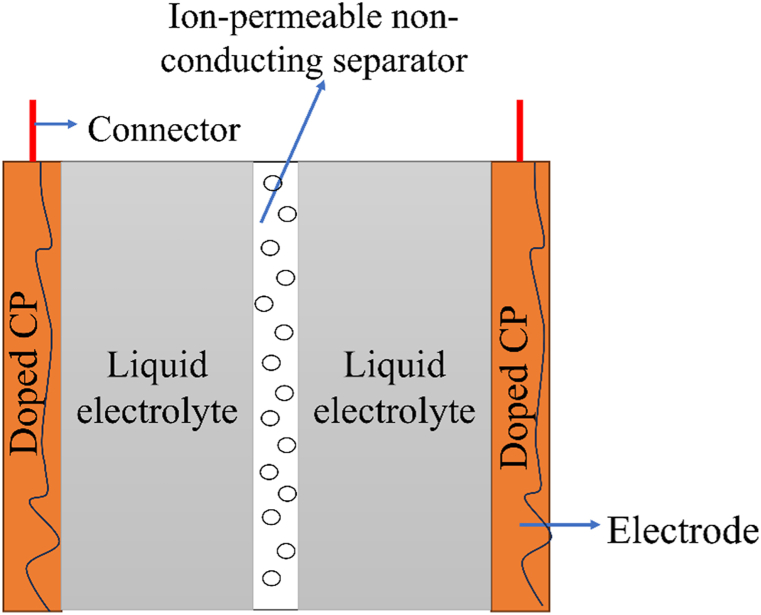
Schematic diagram of supercapacitor.

Supercapacitors, also known as ultracapacitors, are electrochemical energy storage devices with a lower voltage limit than traditional capacitors but with much greater capacitance. They serve as a link between rechargeable batteries and electrolytic capacitors, having energy per unit volume higher than ordinary capacitors but lower than batteries. The electrochemical performance of a supercapacitor is evaluated through some parameters, such as the specific capacitance, cycle life, charge-discharge rate, etc. Compared to traditional capacitors, they have a greater cycle life, high specific capacitance, and fast charging-discharging time [160]. Supercapacitors are made up of two electrodes submerged in an electrolytic solution, with a non-reactive, non-conductive, ion-permeable barrier separating them [Fig. 14**]**. They are classified into three classes depending on their charge storage mechanism: electrochemical double layer capacitors (EDLCs), pseudocapacitors, and hybrid supercapacitors. EDLCs are non-faradic supercapacitors in which charge is stored at the electrode-electrolyte interface by electrostatic force of attraction. When an EDLC device is charged, an electrical bilayer-often called Helmholtz double layer-forms at the interface which is why it is called an electrochemical double layer capacitor. On the other hand, a pseudocapacitor works in a faradaic way where charge storage occurs by intercalation or redox reaction between electrode and electrolyte ions. Pseudocapacitors offer more power and charge density compared to EDLCs. On the other hand, a hybrid supercapacitor combines the features of an EDLC and a pseudocapacitor, allowing for both faradaic and non-faradic charge storage. Over the past several years, there has been an increase in interest in the development of conducting polymers as electrode materials for pesudocapacitors. Owing to their favorable characteristics, which include easy processing, quick redox reactivity, high pseudocapacitance, fast charging-discharging ability, suitable morphology, plasticity, film formability, environmental friendliness, lightweight, cheap manufacturing costs, and enhanced flexibility, CPs and their composites are crucial in the creation of economically feasible electrode materials for supercapacitors [[161], [162], [163], [164], [165]]. There are three varieties of conducting polymer-based supercapacitors: type 1 (symmetric) supercapacitors use the same p-doped CPs for both electrodes, type 2 (asymmetric) supercapacitors use two different p-doped CPs for two different electrodes, and type 3 (symmetric) use the same CPs for two electrodes, but in two different doping states: n-dopped for the negative electrodes and p-doped for the positive electrodes [166]. In any case, since volume shrinkage amid the dedoping operations of some CPs decreases their cyclic stability, current research has centered on improving the execution of CPs by utilizing their composite with a metal oxide or carbon-based materials such as graphene oxide (GO). Hence, synergistic impacts have expanded the capacitance capabilities of such progressed cross-breed materials, particularly for supercapacitor applications [167]. Furthermore, a composite of CPs made of materials with a wide surface area increases the amount of space that may be used for charge storage, which raises the capacitance [168]. For instance, a PANI nanocomposite fabricated with 2 % GO having a surface area of 39.595 m^2^/g shows a maximum specific capacitance of 658 Fg^-1^ at a current density of 10Ag^-1^, while pure PANI with a surface area of only 1.998 m^2^/g shows a specific capacitance of only 158 Fg^-1^ at the same current density [169]. Prasankumar et al. synthesized PANI/Fe_3_O_4_ nanocomposite with a surface area of 64.71 m^2^/g which is greater than the surface area of the individual materials. Therefore, an electrode made up of this nanocomposite shows a high specific capacitance of 572 Fg^-1^ at 0.5 Ag^-1^ current density with a cyclic stability retention of 82 % after 5000 cycles at 1 Ag^-1^ in a three-electrode setup system when 1M H_2_SO_4_ is used as an electrolyte. The higher specific capacitance and cyclic stability are due to the synergistic effect of PANI and Fe_3_O_4_ and the high surface area of the nanocomposite [170]. The electrochemical performance of some electrodes made of common conducting polymer-based composites is compiled in Table 1.

**Table 1:** Summary of research on the employment of conjugated polymers in supercapacitors.

Electrode Material	Electrolyte	Maximum Specific Capacitance	Cycling stability	Maximum energy density	Power density at maximum energy density	Reference
GNS/PPY/AgNPs	1 M KCl	450 Fg^-1^ at 0.9 mA/g	92.0 % after 1000 cycles	81.0 Whkg^−1^ at 0.9 mA/g	10.13 kWkg^−1^ 0.9 mA/g	[171]
Ti_3_C_2_/PPy	2 M H_2_SO_4_	109.4 mFcm^−2^ at 1.05 mAcm^−2^	96 % after 10000 cycles	3.398 mW hcm^−2^	0.0845 mW cm^−2^	[172]
PEDOT/g-C_3_N_4_	1 M Na_2_SO_4_	200 Fg^-1^ at 2 A/g	96.5 % after 1000 cycles	17.5 Whkg^−1^	5 Wkg^-1^	[173]
NiCo_2_O_4_-PANI	1 M H_2_SO_4_	901 Fg^-1^ at 1 A/g	91 % after 3000 cycles	81.77 Whkg^−1^	0.399 Wkg^-1^	[174]
MWCNT-COOH/MnO_2_/PANI	1 M KCl	517.13 ± 15.25 Fg^-1^ at 1 mA constant current	90 % after 1000 cycles	71.88 ± 2.12 Whkg^−1^	10.08 ± 0.26 Wkg^-1^	[175]
PDMS/CNTs/PANI	[BMIM][BF_4_]	265 Fg^-1^ at 1 mAcm^−2^	76 % after 5000 cycles	25.5 Wh kg^−1^	126.6 Wkg^-1^	[176]
Fe_3_O_4_/CNT/PANI	PVA-H_2_SO_4_	201 Fg^-1^ at 20 mVs^−1^ scan rate	96.4 % after 10,000 cycles	28 Wh kg^−1^	5.3 kWkg^−1^	[177]

### 5.5 Light emitting diodes

As an alternative to conventional bulbs, light-emitting diodes are becoming popular because of their high efficiency, low energy consumption, compact size, and durability. In addition to the high expense of waste management and disposal, traditional lighting systems also run the danger of catching fire when exposed to the environment [178]. Also, each ordinary electric bulb, including compact fluorescent lamps (CFLs), contains 3–5 mg of mercury, a dangerous heavy metal that can harm the kidneys, liver, and immune system in addition to causing irreversible hematological damage [179] among others. Incandescent bulbs, on the other hand, have around three times the amount of mercury per bulb than CFLs, and if they are disposed of incorrectly, they might seriously harm the environment as well as living organisms.

Because of their long lifespan, great efficiency, low power consumption, and eco-friendliness, Polymer Light-Emitting Diodes (PLEDs), also known as Organic Light-Emitting Diodes (OLEDs), are gaining interest nowadays over conventional lighting systems. These days, the industry offers a wide range of OLED-based products, some of which may eventually be bendable and flexible. These include computer displays, cellphones, digital cameras, MP3 players, flat-screen TVs, and portable gaming consoles. Under the proper electric potential, CPs, which have band gaps that are comparable to those of conventional semiconductors, produce light. This is also referred to as electroluminescence, which has been discussed in the **Optical Properties** [Section 4] section. Because of its excellent optical properties, small band gap energy, highly ordered crystalline thin film formability, and bright yellow fluorescence, poly (p-phenylene vinylene), with emission maxima at 551 nm (2.25 eV) and 520 nm (2.4 eV) [180,181], was the first electroluminescent CP to be employed in the preparation of OLEDs. Besides, other conducting polymers with potential for application in OLEDs include poly (p-phenylenevinylenes), polyanilines, polypyrroles, Poly (dialkylfluorenes), Polythiophenes, Poly (3,4-ethylenedioxythiophene) and their different composites and derivatives [[182], [183], [184], [185], [186], [187], [188], [189], [190], [191]]. Some conducting polymers have multi-colored electrochromic states depending on their oxidation states [192] and therefore gather interest in a wide variety of optoelectronic applications. These days, quick switching speeds, low driving potential, high coloring efficiency, high contrast, and multi-colored electrochromic properties of CPs are utilized in low-power electrochromic displays and windows [[193], [194], [195], [196]].

### 5.6 Anticorrosion coatings

When metal surfaces are exposed to air, moisture, an alkaline or acidic environment, or both, a natural process known as corrosion occurs, which converts the metals into undesirable substances. Anti-corrosion coatings shield metal parts against deterioration brought on by moisture, salt spray, alkaline or acidic environment, oxidation, or exposure to a range of industrial or environmental pollutants. These days, there are several methods and coatings available for preventing corrosion. Primers, topcoats, and conversion coatings are the three categories into which corrosion inhibition coatings have been classified. The general process for creating inhibitory coatings involves adding pigments that release substances that prevent corrosion and protect metal surfaces by absorbing moisture. The inhibitory qualities of chromate ions are well-known. Because of their superior corrosion prevention qualities, chromate-based corrosion-inhibiting primer coatings have been in use for a very long time. Today, however, their usage is restricted due to the carcinogenic and poisonous effects of hexavalent chromium ions present in dichromate. In addition, conventional anticorrosion coatings made of metal have limitations since they leak toxic inhibitors into the environment on a regular basis, are expensive, and have a limited lifespan [197,198]. Conventional coatings based on organic compounds that work as a boundary to ionic species, oxygen, and water development, such as silicone, epoxy, fluorocarbon, polyurethane, and zinc-rich coatings, lower the rate of metal corrosion. These coatings do, in any case, have a few genuine deficiencies, such as constrained toughness, weak bond strength, destitute weatherability, and low impact strength [[199], [200], [201]]. Also, epoxy and polyurethane-based coatings are found to release bisphenol-A and other toxicity to the aquatic environment and food chain [202,203]. Conducting polymer-based pigmented paint coatings and their composites are becoming more and more popular as an alternative to conventional anticorrosion coatings because of their facile synthesis, high conductivity, high stability, eco-friendliness, economic viability, and special redox characteristics [[204], [205], [206]]. Furthermore, CP-based coating materials offer superior anticorrosion properties than standard coating materials because they may function as an electrical and physical barrier whereas traditional coating materials only provide a physical barrier between the metal and the environment [207]. To account for the anticorrosion properties of CPs, several ideas have been proposed. The anodic protection mechanism, the physical barrier effect mechanism, and controlled inhibitor release are the three main theories put out to explain the anticorrosion features of CPs.

1. Anodic protection theory: This theory states that CP, being an oxidant, creates a metal oxide layer between the coating and the metal surface because of its superior oxidative properties. Because of these layers of metal oxide, the metal is protected from the atmosphere, and corrosion is therefore prevented [[208], [209], [210], [211], [212], [213]].
2. The physical barrier effect: According to the barrier effect, the polymer coating shields the substrate metals from corrosion by acting as a barrier to keep hostile anions, salts, and oxidants from the environment from penetrating, therefore shielding the metal from being corroded [214].
3. Controlled inhibitor release mechanism: According to this concept, anion-doped oxidized CP-based coatings deposited on a metal surface have the ability of controlled release of the anion upon reduction resulting from coupling to the substrate metal through defects in the coating. Consequently, the release of the corrosion inhibitor is caused by the defects in the coating, which makes it an excellent corrosion-inhibiting coating [204,208].

Research utilizing potentiodynamic polarization and electrochemical impedance spectroscopy demonstrates that PANI effectively prevents corrosion of aluminum in a 3.5 % NaCl solution. The corrosion current of uncoated aluminum is significantly reduced from 6.55 μA cm^−2^ to 0.158 μA cm^−2^ when coated with PANI. The corrosion rate of the aluminum coated with PANI is measured at 5.17 × 10^−4^ mm per year, which is about 40 times lower than that of uncoated aluminum. Moreover, the corrosion potential shifts from −1.015 V versus SCE for uncoated aluminum to approximately −0.9 V versus SCE for the polyaniline-coated aluminum. This positive shift of about 0.11 V indicates that the PANI coating offers substantial protection to the aluminum surface [215].

Mobin et al. [216] developed a ternary nanocomposite of polythiophene, titanium dioxide, and reduced graphene oxide (PTh-TiO_2_-rGO) through oxidative polymerization with FeCl_3_. This composite, when applied to low-carbon steel, demonstrates superior anticorrosion properties. With a coating thickness of 14.9 ± 1 μm, it outperforms pure PTh and PTh-TiO_2_ coatings in a 3.5 wt % NaCl solution, exhibiting the lowest corrosion current density (*Icorr* = 0.570 × 10-6 A cm-2), highest positive shift in corrosion potential (*E*_corr_ = −0.578 V), the highest impedance and phase angle values (3.56 × 10^3^ Ω cm^2^), enhanced hydrophobicity (contact angle of 94°), and a protection efficiency of 99 %. Table 2 provides an overview of research on the application of conducting polymers for preventing corrosion in metals.

**Table 2:** An overview of studies on the use of conducting polymers in corrosion inhibition applications.

Substrate	Coating Material	Medium	Thickness (μm)	Maximum Efficiency	References
Stainless steel	PPy-SMF	3.5 % NaCl	–	99 %	[217]
Iron	PANI/epoxy/Zn	1 M HCl	70 ± 5	–	[218]
Iron	PANI/TiO_2_	3 % NaCl	100 ± 5	–	[219]
Low carbon steel	PTh/TiO_2_/RGO	3.5 % NaCl	14.9 ± 1	99 %	[220]
Aluminum alloy	PANI	3.5 % NaCl	–	90 %	[221]
Carbon steel	PANI/PVC	3 % NaCl in 0.1 M HCl	19 ± 1	–	[222]
Mild steel	PANI dopped with BS and LS	3.5 % NaCl	50 ± 5	–	[223]

### 5.7 Waste water treatment

**Fig. 15:**
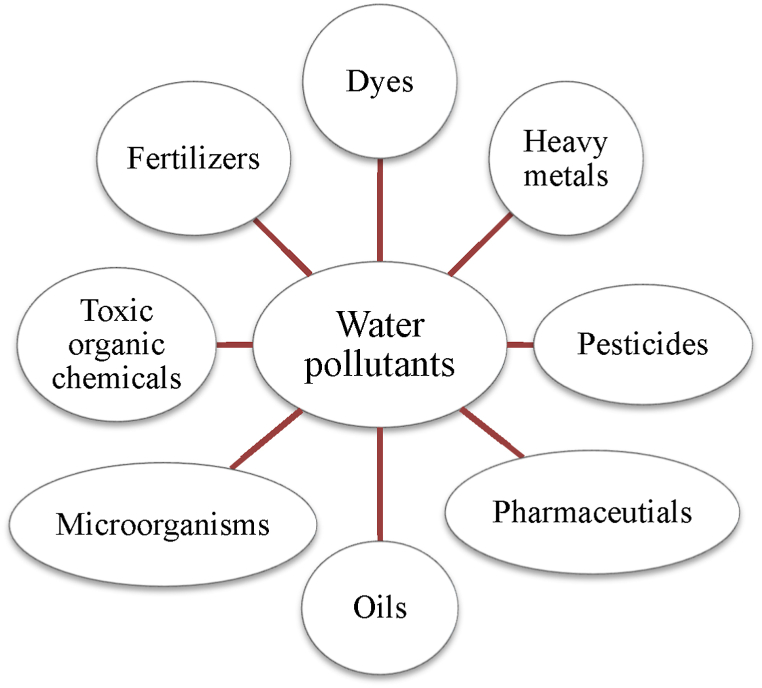
Sources of water pollution.

Rapid industrialization and globalization have caused major environmental pollution, with water pollution being especially severe and greatly affecting the availability of clean water [18,[224], [225], [226], [227]]. The principal pollutants in water include fertilizers, pesticides, oils, pharmaceutical wastes, synthetic organic dyes used in the textile industries, and heavy metals produced by a variety of industries, including metal plating, tanneries, and batteries [Fig. 15**]**. Among the human endeavors that use water the most and contaminate water sources is the textile industry. Global textile expansion leads to an annual release of about 280,000 tons of non-biodegradable dyes into receiving water bodies, endangering ecological systems and public health by decreasing DO levels, increasing BOD and COD levels, hindering photosynthesis, altering natural salinity of water, impeding plant growth, entering the food chain, causing recalcitrance and bioaccumulation, and possibly promoting toxicity, dermatitis, skin irritation, mutagenicity, and carcinogenicity [[228], [229], [230], [231], [232], [233]]. In addition to the textile industry, other industries that leak dyes into the environment include paper, plastic, rubber, concrete, cosmetics, and paint [232,233]. Pesticides are a significant category of water pollutants, employed to enhance agricultural productivity and food production by protecting crops from weeds, diseases, and pests. Therefore, to supply the world's expanding population with food, the use of pesticides in agriculture is rising progressively. But the worrying thing is that, even though only 1 % of the pesticides used are effective at controlling pests, the remainder contaminate soil and water by mixing with them, upsetting the ecosystem's delicate balance and posing a risk to human health by getting into the food chain [234]. Apart from textile dyes, pesticides, heavy metals, and their ions, other pollutants mentioned earlier pose a threat to human beings, aquatic species, and ecosystems. Therefore, it is essential to treat wastewater adequately to lessen its harmful consequences.

**Scheme 8:**
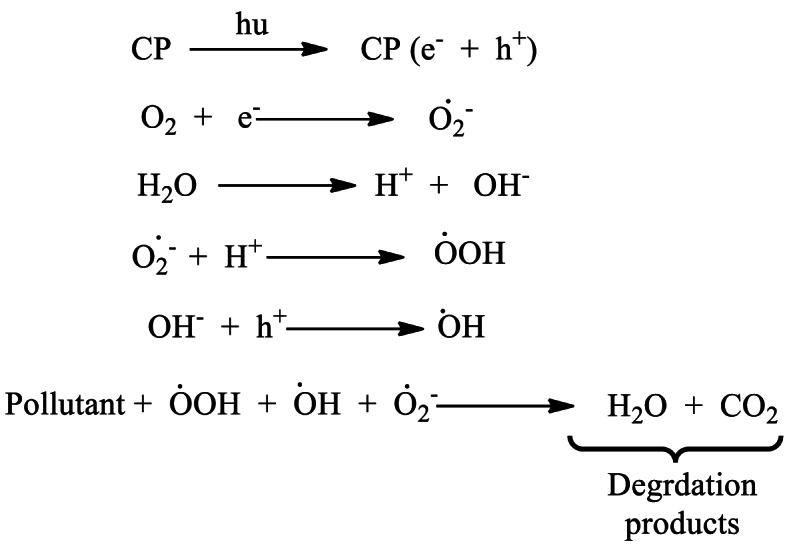
Reactions involved in photocatalysis.

**Fig. 16:**
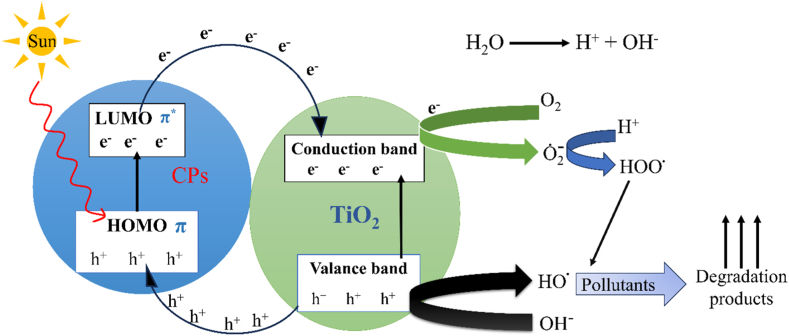
Mechanism in CP-based composite for the degradation of textile dyes.

Photocatalytic degradation in the presence of sunlight is considered a safe, economical, and environmentally beneficial method of removing hazardous materials from wastewater. It has been investigated that metal oxide-based semiconductors (TiO_2_, SnO_2_, CeO_2_, ZrO_2_, WO_3_, and ZnO) are good photocatalysts for the effective degradation of organic contaminants in wastewater. However, because of their large band gap energies (UV range) and the length of time it takes for photogenerated electron-hole pairs to recombine, their photocatalytic activity is often constrained [235]. In any case, the photocatalytic action of those metal oxides can be progressed by forming composites with conducting organic polymers. Photocatalytic degradation of water pollutants involves the irradiation of the CP-metal oxide composite by a light source (usually sunlight), which causes the excitation of electrons from the π to π∗ orbital of the CP. In the case of such composites, CPs act as a photosensitizer to absorb a wide range of photons because of the lower bandgap compared to metal oxide. Upon the absorption of photons having energy greater than or equal to the band gap of the semiconductor, electrons (e^−^) are promoted to the LUMO, thereby creating holes (h^+^) in the HOMO of the CP. The photogenerated electrons possess energy levels comparable to the conduction band of the metal oxide, facilitating their migration from the highest occupied molecular orbital (HOMO) of the conjugated polymer to the valence band of the metal oxide. These photoinduced electrons finally transfer to the surface of the metal oxide and react with oxygen and water to produce hydroxyl and superoxide anion radicals, which degrade water pollutants upon oxidation. The migration of photoinduced electrons from the LUMO of CP to the CB of metal oxide causes the generation of holes in the VB of metal oxide, which finally transfer to the surface of the metal oxide and cause degradation of contaminants upon generation of hydroxyl free radical [Scheme 8]. As the holes in the VB of metal oxide has the equivalent energy as the HOMO of CP, these holes finally migrate to the HOMO of CP [Fig. 16]. Saravanan et al. have synthesizd PANI-ZnO nanoomposite to study the photocatalytic effect of pure ZnO and PANI-ZnO nanocomposite [236]. The incorporation of PANI in ZnO decrease the band gap of pristine ZnO and increases the photocatalytic activity by 15–20 times. The synthesized nanocomposite has a maximum degradation efficiency of 98.3 % and 99.25 % for methyl orange dye and methylene blue dye respectively after 180 min of exposure to this photocatalyst, while pristine ZnO exhibited only 4.6 and 6.6 efficiency respectively. In addition to metal oxide-based composites, graphene oxide (GO) and reduced graphene oxide (RGO) based composites have also gathered much interest in recent days because of their high surface area, outstanding physical and chemical properties, tunable band gap energies and broad range of light absorption capabilities [[237], [238], [239], [240], [241]]. The photocatalytic behavior of PANI-reduced graphene oxide composite was investigated by Mitra et al. [242]. This PANI-RGO composite, with 5 wt % RGO, shows excellent visible-light-driven photocatalytic activity to remove organic dye from water with degradation percentages of 99.68, 99.35, and 98.73 for malachite green, rhodamine B, and congo red within 15, 30, and 40 min, respectively. The improved photocatalytic activity is attributed to better electron-hole pair separation and a reduced band gap (2.74 eV for the PANI-RGO composite compared to 2.95 eV for pure PANI). The PANI/RGO composite also demonstrated remarkably high photostability even after six cycles ((99.68−86.63 % for methylene blue), (99.35−87.12 % for rhodamine B), and (98.73−92.78 % for congo red)). Table 3 provides an overview of research on the use of conducting polymer-based composite in photocatalytic wastewater treatment.

**Table 3:** An overview of research on conducting polymer-based composites for photocatalytic wastewater treatment.

Composite (Photocatalyst)	Light Source	Pollutant	Efficiency	Time	Reference
Polyaniline/TiO_2_	UV lamp	Bisphenol-A	99.7 %	80 min	[243]
Polyaniline/NiO	Tungsten lamp (visible light)	Methylene Blue	76 %	5 h	[244]
Polyaniline/TiO_2_	Visible	Methylene Blue	81.74 %	2 h	[245]
Polyaniline/FeO-ZnO	Visible light	3-aminophenol	92 %	2 h	[246]
Polypyrrole/TiO_2_	Simulated sunlight	Methyl orange	90 %	2 h	[247]
Polypyrrole/MOSe_2_	Visible light	Congo Red	84.4 %	15 min	[248]
		Rhodamine B	98.5 %		
PEDOT/MnO_2_	Sunlight	Methylene Blue	95.7 %	7 h	[249]
Polythiophene/MnO_2_	Visible light	Malachite green	98 %	80 min	[250]
Polythiophene/TiO_2_	UV light	Rhodamine B	76 %	3 h	[251]
			98 %	10 h	

**Fig. 17:**
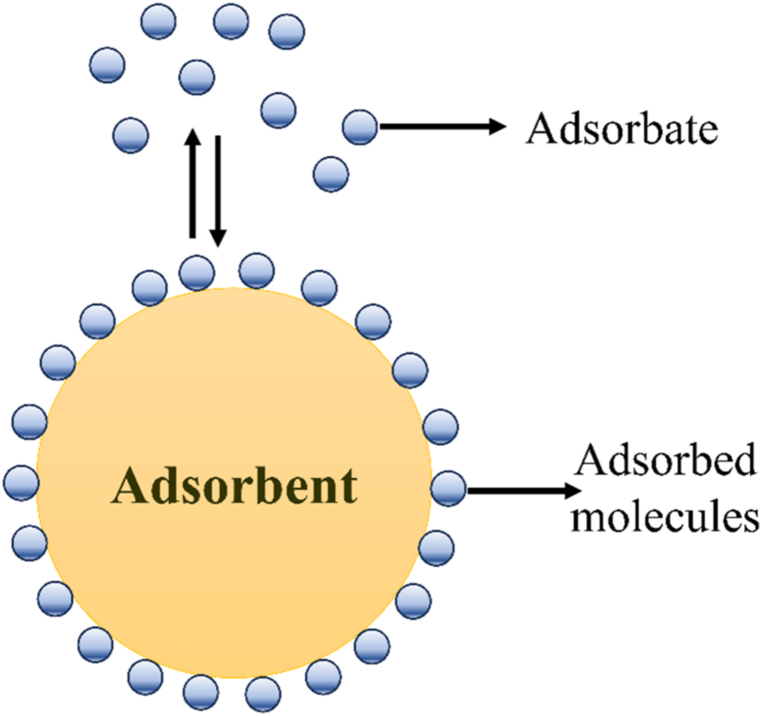
Illustration of the adsorbent, adsorbate, and adsorption processes.

One of the fastest, most economical, and most efficient methods for decontaminating water without creating any byproducts is adsorption. It is a surface phenomenon that happens when an atomic or molecular layer of a solute (gas or liquid) called adsorbate builds up on the surface of an adsorbent (usually solid), and the adsorbate in contact with the adsorbed species is in a state of dynamic equilibrium **[**Fig. 17]. Heavy metal ions are extremely detrimental to living organisms, even at very low concentrations. At this low concentration, conventional methods such as precipitation cannot effectively remove them from wastewater. The ability of adsorption process to eliminate traces of heavy metal ions from wastewater is perhaps its most intriguing feature [252].

**Scheme 9:**
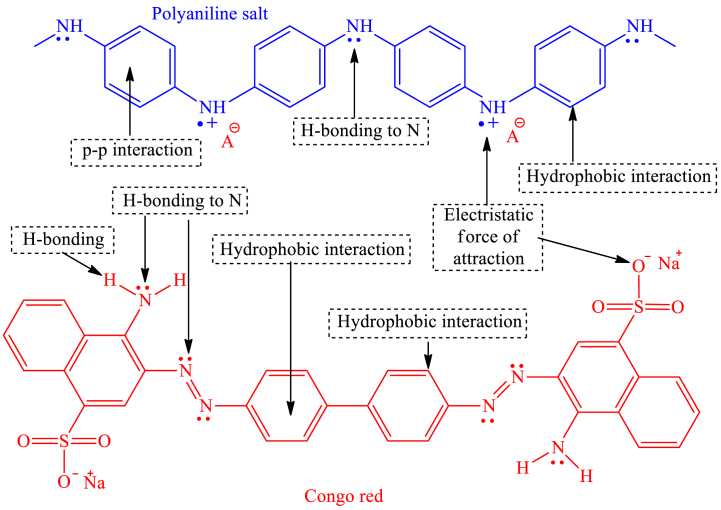
Possible interactions between polyaniline and an organic dye (Congo red).

Composites of CPs may efficiently remove pollutants from water through an adsorption process in addition to photocatalytic degradation. Apart from their ability to degrade water pollutants through photocatalysis, conducting polymers are found to exhibit excellent adsorption properties due to the presence of easily adjustable functional groups, π-bonds, and atoms such as N, O, P, and S on their backbone [[253], [254], [255]]. Electrostatic attraction, π-bonding interaction, hydrophobic interactions, chelation, and ion exchange are possible mechanisms [Scheme 9**]** that are responsible for improved adsorption through the use of conducting polymers [256]. For the adsorption of several contaminants, including heavy metals and dyes, PANI is the conducting polymer that has been researched the most [257]. Wang et al. [258] synthesized a polyaniline-modified TiO_2_ (PANI/TiO_2_) composite to study Acid red adsorption. The composite demonstrated high adsorption efficiency with an adsorption-desorption equilibrium time of about 5 min and a maximum adsorption capacity of 454.55 mg/g. The study revealed that the adsorption of acid red onto PANI/TiO_2_ followed the pseudo-second-order kinetics. Additionally, the process reached an adsorption-desorption equilibrium that conformed to the Langmuir adsorption isotherm, indicating that the adsorption occurred as a single layer. Table 4 summarizes an overview of research on the use of CP-based composites in the removal of water contaminants by adsorption mechanism.

**Table 4:** Summary of research on the use of conductive polymer-based composite in adsorption-based removal of pollutant from waste water.

Composite (Adsorbent)	Adsorbate (Pollutant)	Efficiency (mg/g)	Isotherm	Reference
Carboxymethyl tragacanth gum-grafted-polyaniline/Fe_2_O_3_	Amoxicillin (antibiotic)	909.09	Freundlich Isotherm	[259]
Polypyrrole/Zeolite	Rhodamine B	86.2 % removal from 75 mg/l solution by 1.8 g/l composite	Freundlich Isotherm	[260]
	Reactive Red	88.3 % removal from 75 mg/l solution by 1.8 g/l composite		
Polythiophene/Zeolite/Fe	Methylene Blue	319.4	Toth Isotherm	[261]
Polyaniline/CuFe	Methyl Orange	345.9	Freundlich Isotherm	[262]
Polythiophene/TiO_2_	Pb^2+^	82.10	N/A	[263]
	Zn^2+^	30.72		
	Cu^2+^	60.79		
C-Polypyrrole/MoO_2_	Pb^2+^	381.87	N/A	[264]
Co_3_O_4_/Polypyrrole/Graphene	Methylene Blue	663.018	Langmuir & Temkin Isotherm	[265]
	Congo Red	659.056		
	Pb^2+^	780.363		
	Cd^2+^	794.188		
Polypyrrole/Monodisperse Latex Spheres	Cr^6+^	343.64	Langmuir Isotherm	[266]

### 5.8 Other applications

In addition to their traditional uses, conducting polymers are increasingly being explored for a variety of advanced uses. These include electronic nano-devices, electrodes, sensors, field-effect transistors, drug delivery systems, tissue engineering, and catalysis, among many others [[267], [268], [269]].

## 6 Challenges and future prospects

Because conducting organic polymers (CPs) and their composites have a unique mix of electrical conductivity, mechanical flexibility, processability, and environmental friendliness, they present a versatile class of materials with a broad range of potential applications in many disciplines. Despite having numerous advantages and a wide range of applications, CPs also come with certain limitations. Their limited electrical conductivity compared to conventional metals and semiconductors limits their use in electronic devices that necessitate materials with higher electrical conductivity. Aside from this, some CPs, such as polyacetylene, suffer from low processability and poor mechanical properties, which further limit their application. Also, most of the conducting polymers require doping to increase their conductivity. Doping often produces salts of CPs with reduced solubility, which are more difficult to process. Many conducting polymers can eventually lose their mechanical and electrical qualities when exposed to environmental elements such as oxygen, moisture, and UV light. It is still difficult to increase the stability of these materials in practical conditions. Continued research focused on enhancing their performance, stability, biocompatibility, synthesis, composite formation, and processing is necessary to overcome these constraints. Addressing these challenges will be crucial for unlocking the full potential of conducting polymers across various fields and broadening their applications.

Researchers at IMDEA Nanoscience in Madrid, Spain, have recently shown how a 1D linearly bridged acene polymer with narrow bandgap energy in-gap zero energy edge states can undergo a quantum phase transition from the topologically trivial to non-trivial class [270]. These polymers offer great promise for near-infrared applications and can be employed in systems that need to have high electrical conductivity to fulfill future demands.

The conducting organic polymers that have been most thoroughly researched are polyaniline, polypyrrole, polythiophenes, and poly (3,4-ethylenedioxythiophene). To fully exploit and explore the possibilities of other prospective CPs in this field, an increasing amount of research needs to be done on them. Additionally, future studies should emphasize cost-effective synthesis and device development to achieve successful commercialization.

## 7 Concluding remarks

Due to their tunable electronic and optical properties, as well as their ease of synthesis and processing, conducting polymers-which mainly include polyaniline, polypyrrole, polythiophenes, and poly (3,4-ethylenedioxythiophene)- find applications in a wide range of industries, including photovoltaic cells, LEDs, OLEDs, batteries, anticorrosion coatings, wastewater treatment, supercapacitors, transistors, and electronic nanodevices. Therefore, CPs have become a center of interest to the researcher for the past few decades. This review article effectively demonstrated an in-depth concept and applications of conducting polymers.

The following points are a brief summary of some important aspects of this review article.

• In their pristine state, conducting polymers are either poor electrical conductors or insulators. However, doping with particular doping agents can significantly increase their conductivity. These chemicals enhance the electrical conductivity of the polymer by either donating electrons (n-doping) to or withdrawing electrons (p-doping) from the π-conjugated system.
• Conventional band theory cannot adequately explain the high electrical conductivity of doped CPs. Doping generates quasi-one-dimensional charge carriers like solitons, polarons, and bipolarons along the π-conjugated polymer backbone. These charge carriers facilitate the electrical transition from the VB to the CB by creating soliton or bipolaron bands, which narrow the band gap energy, thereby improving conductivity.
• When subjected to the proper electrical potential, CPs emit light, similar to inorganic semiconductors. This property of electroluminescence is utilized in OLEDs. In contrast, organic solar cell devices exploit the photovoltaic effect, where CPs convert light into electrical power. Furthermore, the electrochromic properties of CPs are employed in devices like smart windows and displays, allowing for dynamic color changes and transparency adjustments.
• Conducting polymers are also utilized in energy storage systems like supercapacitors and rechargeable batteries. However, more studies and development are required to advance the technology of conducting polymer-based batteries.
• Using conducting polymers in electronic devices could potentially replace heavy metals, thereby helping to reduce heavy metal pollution. CPs can further contribute to pollution control through their ability to treat wastewater by both adsorption and photocatalytic mechanism.

## CRediT authorship contribution statement

**Md. Byzed Hasan:** Writing – original draft, Formal analysis, Data curation. **Md. Masud Parvez:** Conceptualization, Supervision, Writing – review & editing. **Md. Abrar Yasir Abir:** Writing – review & editing. **Md. Faruak Ahmad:** Writing – review & editing.

## Declarations

This manuscript has not been published or presented elsewhere, either in part or in full. It is also not under consideration for publication in any other journal. We have reviewed and fully adhere to the policies of your journal, and we confirm that neither the manuscript nor the study infringes upon any of these guidelines.

## Ethics approval

There are no studies by any of the authors in this article that used human subjects.

## Fundings

This research did not receive any external funding.

## Declaration of competing interest

The authors declare that they have no known competing financial interests or personal relationships that could have appeared to influence the work reported in this paper.

## References

[1] Bahrani S., Hashemi S.A., Mousavi S.M., Arjmand M., Ghalamfarsa F., Ghaedi M. (2022). ACS Symposium Series, American Chemical Society.

[2] Das T.K., Prusty S. (2012). Review on conducting polymers and their applications. Polym. Plast. Technol. Eng..

[3] Matula R.A. (1980). Electrical resistivity of copper, goid r palladium, and silver’. http://jpcrd.aip.org/about/rights_and_permissions.

[4] Letheby H. (1862). On the production of a blue substance by the electrolysis of sulphate of aniline. J. Chem. Soc..

[5] Shirakawa H., Louis E.J., MacDiarmid A.G., Chiang C.K., Heeger A.J. (1977). Synthesis of electrically conducting organic polymers: halogen derivatives of polyacetylene, (CH)x. J. Chem. Soc. Chem. Commun..

[6] Cairns C.N. (2005).

[7] Bhuie A.K., Ogunseitan O.A., Saphores J.D.M., Shapiro A.A. (2004). IEEE International Symposium on Electronics and the Environment.

[8] Rautela R., Arya S., Vishwakarma S., Lee J., Kim K.H., Kumar S. (2021). E-waste management and its effects on the environment and human health. Sci. Total Environ..

[9] Xu X., Zeng X., Boezen H.M., Huo X. (2015). E-waste environmental contamination and harm to public health in China. Front. Med..

[10] Mittal A., Naushad Mu, Sharma G., Alothman Z.A., Wabaidur S.M., Alam M. (2016). Fabrication of MWCNTs/ThO _2_ nanocomposite and its adsorption behavior for the removal of Pb(II) metal from aqueous medium. Desalination Water Treat..

[11] Alqadami A.A., Khan M.A., Siddiqui M.R., Alothman Z.A. (2018). Development of citric anhydride anchored mesoporous MOF through post synthesis modification to sequester potentially toxic lead (II) from water. Microporous Mesoporous Mater..

[12] Chakraborty S.C., Qamruzzaman M., Zaman M.W.U., Alam M.M., Hossain M.D., Pramanik B.K., Nguyen L.N., Nghiem L.D., Ahmed M.F., Zhou J.L., Mondal M.I.H., Hossain M.A., Johir M.A.H., Ahmed M.B., Sithi J.A., Zargar M., Moni M.A. (2022). Metals in e-waste: occurrence, fate, impacts and remediation technologies. Process Saf. Environ. Protect..

[13] Wiegel S., Aulinger A., Brockmeyer R., Harms H., Löffler J., Reincke H., Schmidt R., Stachel B., Von Tümpling W., Wanke A. (2004). Pharmaceuticals in the river Elbe and its tributaries. Chemosphere.

[14] Quesada H.B., Baptista A.T.A., Cusioli L.F., Seibert D., de Oliveira Bezerra C., Bergamasco R. (2019). Surface water pollution by pharmaceuticals and an alternative of removal by low-cost adsorbents: a review. Chemosphere.

[15] Bouafia A., Meneceur S., Chami S., Laouini S.E., Daoudi H., Legmairi S., Mohammed Mohammed H.A., Aoun N., Menaa F. (2023). Removal of hydrocarbons and heavy metals from petroleum water by modern green nanotechnology methods. Sci. Rep..

[16] Mojiri A., Zhou J.L., Robinson B., Ohashi A., Ozaki N., Kindaichi T., Farraji H., Vakili M. (2020). Pesticides in aquatic environments and their removal by adsorption methods. Chemosphere.

[17] Fernández C., Larrechi M.S., Callao M.P. (2010). An analytical overview of processes for removing organic dyes from wastewater effluents. TrAC, Trends Anal. Chem..

[18] Van Tran T., Nguyen D.T.C., Kumar P.S., Din A.T.M., Qazaq A.S., Vo D.-V.N. (2022). Green synthesis of Mn3O4 nanoparticles using Costus woodsonii flowers extract for effective removal of malachite green dye. Environ. Res..

[19] Alothman Z.A., Wabaidur S.M. (2019). Application of carbon nanotubes in extraction and chromatographic analysis: a review. Arab. J. Chem..

[20] Naushad Mu, Sharma G., Alothman Z.A. (2019). Photodegradation of toxic dye using Gum Arabic-crosslinked-poly(acrylamide)/Ni(OH)2/FeOOH nanocomposites hydrogel. J. Clean. Prod..

[21] Azhar A., Yamauchi Y., Alothman Z.A., Badjah A.Y., Naushad Mu, Habila M., Wabaidur S., Wang J., Zakaria M.B. (2019). Nanoporous iron oxide/carbon composites through in-situ deposition of prussian blue nanoparticles on graphene oxide nanosheets and subsequent thermal treatment for supercapacitor applications. Nanomaterials.

[22] Azam M., Wabaidur S., Khan M., Al-Resayes S., Islam M. (2022). Heavy metal ions removal from aqueous solutions by treated ajwa date pits: kinetic, isotherm, and thermodynamic approach. Polymers.

[23] Kumar N., Kumari M., Ismael M., Tahir M., Sharma R.K., Kumari K., Koduru J.R., Singh P. (2023). Graphitic carbon nitride (g–C3N4)–assisted materials for the detection and remediation of hazardous gases and VOCs. Environ. Res..

[24] Plieth W., Plieth W. (2008). Electrochemistry for Materials Science.

[25] del Río C., Acosta J. (1994). Extrinsic conducting and superconducting polymer systems: 1. Analysis of the structure of PVDF/PS blends containing copper and carbon black fillers. Polymer.

[26] Gorman C.B., Ginsburg E.J., Grubbs R.H. (1993).

[27] (1985). The concept of ‘doping’ of conducting polymers: the role of reduction potentials. Phil. Trans. Roy. Soc. Lond. Math. Phys. Sci..

[28] Naarmann H., Theophilou N. (1987).

[29] Kaneko H., Nogami Y., Ishiguro T., Nishiyama H., Ishimoto H., Takahashi A., Tsukamoto J. (1993). Low temperature electrical conductivity of highly conducting iodine doped polyacetylene. Synth. Met..

[30] Tsukamoto J., Takahashi A. (1991). Synthesis and electrical properties of polyacetylene yielding conductivity of 105 S/cm. Synth. Met..

[31] T. Granier, E.L. Thomas, D.R. Gagnon, F.E. Karasz, R.W. Lenz, Structure Investigation of Poly(P-Phenylene Vinylene), n.d.

[32] Ahlskog M., Noguchi T., Ohnishi T. (1997).

[33] Gagnon D.R., Capistran J.D., Karasz F.E., Lenz R.W., Antount S. (1987).

[34] Letheby H. (1862). On the production of a blue substance by the electrolysis of sulphate of aniline. J. Chem. Soc..

[35] Macdiarmid A.G., Chiang J.-C., Halpern M., Huang W.-S., Mu S.-L., Nanaxakkara L.D., Wu S.W., Yaniger S.I. (1985). “Polyaniline”: interconversion of metallic and insulating forms. Mol. Cryst. Liq. Cryst..

[36] Masters J.G., Sun Y., Macdiarmid A.G., Epstein A.J. (1991).

[37] Huang W.-S., Humphrey B.D., MacDiarmid A.G. (1986). Polyaniline, a novel conducting polymer. Morphology and chemistry of its oxidation and reduction in aqueous electrolytes, Journal of the Chemical Society, Faraday Transactions 1. Physical Chemistry in Condensed Phases.

[38] Mottaghitalab V., Xi B., Spinks G.M., Wallace G.G. (2006). Polyaniline fibres containing single walled carbon nanotubes: enhanced performance artificial muscles. Synth. Met..

[39] Kang E. (1998). Polyaniline: a polymer with many interesting intrinsic redox states. Prog. Polym. Sci..

[40] Feast W.J., Tsibouklis J., Pouwer K.L., Groenendaal L., Meijer E.W. (1996). Synthesis, processing and material properties of conjugated polymers. Polymer.

[41] MacDiarmid A.G. (2001). “Synthetic metals”: a novel role for organic polymers (Nobel lecture). Angew. Chem. Int. Ed..

[42] MacDiarmid A.G., Epstein A.J. (1989). Polyanilines: a novel class of conducting polymers. Faraday Discuss. Chem. Soc..

[43] Chiang J.-C., Macdiarmid A.G. (1986).

[44] Macdiarmid A.G., Chiang J.C., Richter A.F., Epstein A.J. (1987). Polyaniline: a new concept in conducting polymers. Synth. Met..

[45] Kulkarni V.G., Campbell L.D., Mathew W.R. (1989). Thermal stability of polyaniline. Synth. Met..

[46] Rapi S., Bocchi V., Gardini G.P. (1988). Conducting polypyrrole by chemical synthesis in water. Synth. Met..

[47] Sabouraud G., Sadki S., Brodie N. (2000). The mechanisms of pyrrole electropolymerization. Chem. Soc. Rev..

[48] Rasmussen S.C. (2015). Early history of polypyrrole: the first conducting organic polymer. https://www.researchgate.net/publication/271530619.

[49] Rasmussen S.C. (2011).

[50] Bolto B., McNeill R., Weiss D. (1963). Electronic conduction in polymers. III. Electronic properties of polypyrrole. Aust. J. Chem..

[51] McNeill R., Siudak R., Wardlaw J., Weiss D. (1963). Electronic conduction in polymers. I. The chemical structure of polypyrrole. Aust. J. Chem..

[52] Bolto B., Weiss D. (1963). Electronic conduction in polymers. II. The electrochemical reduction of polypyrrole at controlled potential. Aust. J. Chem..

[53] Diaz A.F., Kanazawa K.K., Gardini G.P. (1979). Electrochemical polymerization of pyrrole. J. Chem. Soc. Chem. Commun..

[54] Keiji Kanazawa K., Diaz A.F., Gill W.D., Grant P.M., Street G.B., Piero Gardini G., Kwak J.F. (1980). Polypyrrole: an electrochemically synthesized conducting organic polymer. Synth. Met..

[55] Kanazawa K.K., Diaz A.F., Geiss R.H., Gill W.D., Kwak J.F., Logan J.A., Rabolt J.F., Street G.B. (1979). ‘Organic metals’: polypyrrole, a stable synthetic ‘metallic’ polymer. J. Chem. Soc., Chem. Commun..

[56] Diaz A.F., Martinez A., Kanazawa K.K., Salmón M. (1981). Electrochemistry of some substituted pyrroles. J. Electroanal. Chem. Interfacial Electrochem..

[57] Yamaura M., Hagiwara T., Iwata K. (1988). Enhancement of electrical conductivity of polypyrrole film by stretching: counter ion effect. Synth. Met..

[58] Kang H.C., Geckeler K.E. (2000). Enhanced electrical conductivity of polypyrrole prepared by chemical oxidative polymerization: effect of the preparation technique and polymer additive. Polymer.

[59] Osagawara M., Funahashi K., Demura T., Hagiwara T., Iwata K. (1986). Enhancement of electrical conductivity of polypyrrole by stretching. Synth. Met..

[60] Kassim A., Basar Z.B., Mahmud H.N.M.E. (2002). Effects of preparation temperature on the conductivity of polypyrrole conducting polymer. J. Chem. Sci..

[61] Kaloni T.P., Giesbrecht P.K., Schreckenbach G., Freund M.S. (2017). Polythiophene: from fundamental perspectives to applications. Chem. Mater..

[62] Mehmood U., Al-Ahmed A., Hussein I.A. (2016). Review on recent advances in polythiophene based photovoltaic devices. Renew. Sustain. Energy Rev..

[63] McQuade D.T., Pullen A.E., Swager T.M. (2000). Conjugated polymer-based chemical sensors. Chem Rev.

[64] Nielsen C.B., McCulloch I. (2013). Recent advances in transistor performance of polythiophenes. Prog. Polym. Sci..

[65] Mastragostino M., Soddu L. (1990). Electrochemical characterization of “n” doped polyheterocyclic conducting polymers—I. Polybithiophene. Electrochim. Acta.

[66] Österholm J.-E., Passiniemi P., Isotalo H., Stubb H. (1987). Synthesis and properties of FeCl4-doped polythiophene. Synth. Met..

[67] Kaneto K., Yoshino K., Inuishi Y. (1983). Electrical and optical properties of polythiophene prepared by electrochemical polymerization. Solid State Commun..

[68] McCullough R.D., Tristram-Nagle S., Williams S.P., Lowe R.D., Jayaraman M. (1993). Self-orienting head-to-tail poly(3-alkylthiophenes): new insights on structure-property relationships in conducting polymers. J. Am. Chem. Soc..

[69] Gueye M.N., Carella A., Massonnet N., Yvenou E., Brenet S., Faure-Vincent J., Pouget S., Rieutord F., Okuno H., Benayad A., Demadrille R., Simonato J.-P. (2016). Structure and dopant engineering in PEDOT thin films: practical tools for a dramatic conductivity enhancement. Chem. Mater..

[70] Dietrich M., Heinze J., Heywang G., Jonas F. (1994). Electrochemical and spectroscopic characterization of polyalkylenedioxythiophenes. J. Electroanal. Chem..

[71] Heywang G., Jonas F. (1992). Poly(alkylenedioxythiophene)s—new, very stable conducting polymers. Adv. Mater..

[72] Wudl F., Kobayashi M., Heeger A.J. (1984). Poly(isothianaphthene). J. Org. Chem..

[73] Abdulkarim A., Hinkel F., Jänsch D., Freudenberg J., Golling F.E., Müllen K. (2016). A New Solution to an Old Problem: Synthesis of Unsubstituted Poly(*para* -phenylene). J. Am. Chem. Soc..

[74] Remmers M., Müller B., Martin K., Räder H.-J., Köhler W. (1999). Poly(*p* -phenylene)s. Synthesis, optical properties, and quantitative analysis with HPLC and MALDI−TOF mass spectrometry. Macromolecules.

[75] Pavlović D., Cohen S. (2020). Controlled synthesis of unsubstituted high molecular weight poly(*para* -phenylene) *via* Suzuki polycondensation-thermal aromatization methodology. Polym. Chem..

[76] Goldenberg L.M., Lacaze P.C. (1993). Anodic synthesis of poly(p-phenylene). Synth. Met..

[77] Li C., Shi G., Liang Y., Ye W., Sha Z. (1997). High-quality poly(p-phenylene) film prepared by electrochemical polymerization of benzene at a stainless steel electrode. Polymer.

[78] Kovacic Peter, Jones M.B. (1987). Dehydro coupling of aromatic nuclei by catalyst-oxidant systems: poly(p-phenylene). Chem Rev.

[79] Plocharski J. (2000). Mixed conductivity in poly(p-phenylene) doped with iron chloride. Solid State Ion.

[80] Ivory D.M., Miller G.G., Sowa J.M., Shacklette L.W., Chance R.R., Baughman R.H. (1979). Highly conducting charge-transfer complexes of poly(*p* -phenylene). J. Chem. Phys..

[81] Shacklette L.W., Chance R.R., Ivory D.M., Miller G.G., Baughman R.H. (1980). Electrical and optical properties of highly conducting charge-transfer complexes of poly(p-phenylene). Synth. Met..

[82] Shacklette L.W., Elsenbaumer R.L., Chance R.R., Eckhardt H., Frommer J.E., Baughman R.H. (1981). Conducting complexes of polyphenylene sulfides. J. Chem. Phys..

[83] Miyatake K., Iyotani H., Yamamoto K., Tsuchida E. (1996). Synthesis of Poly(phenylene sulfide sulfonic acid) via Poly(sulfonium cation) as a Thermostable Proton-Conducting Polymer. Macromolecules.

[84] Xie L.-H., Yin C.-R., Lai W.-Y., Fan Q.-L., Huang W. (2012). Polyfluorene-based semiconductors combined with various periodic table elements for organic electronics. Prog. Polym. Sci..

[85] Waltman R.J., Bargon J. (1985). The electropolymerization of polycyclic hydrocarbons: substituent effects and reactivity/structure correlations. J. Electroanal. Chem. Interfacial Electrochem..

[86] U. Scherf, E.J.W. List, Semiconducting PolyfluorenesÐTowards Reliable Structure±Property Relationships∗∗, n.d.

[87] Pipertzis A., Papamokos G., Sachnik O., Allard S., Scherf U., Floudas G. (2021). Ionic conductivity in polyfluorene-based diblock copolymers comprising nanodomains of a polymerized ionic liquid and a solid polymer electrolyte doped with LiTFSI. Macromolecules.

[88] Bundgaard E., Krebs F. (2007). Low band gap polymers for organic photovoltaics. Sol. Energy Mater. Sol. Cell..

[89] Pei Q. (1996). Yang, efficient photoluminescence and electroluminescence from a soluble polyfluorene. J. Am. Chem. Soc..

[90] Sirringhaus H., Wilson R.J., Friend R.H., Inbasekaran M., Wu W., Woo E.P., Grell M., Bradley D.D.C. (2000). Mobility enhancement in conjugated polymer field-effect transistors through chain alignment in a liquid-crystalline phase. Appl. Phys. Lett..

[91] Lee K., Maisel K., Rouillard J.-M., Gulari E., Kim J. (2008). Sensitive and selective label-free DNA detection by conjugated polymer-based microarrays and intercalating dye. Chem. Mater..

[92] N. Her, D. Ber, T. of Superconductivity, J. BarnzzN, L.N. Coopzzg Ann J R Scrrzrzzzzzf, PH YSI CAL REVIEW, n.d.

[93] Little W.A. (1964). Possibility of synthesizing an organic superconductor. Phys. Rev..

[94] Labes M.M., Love P., Nichols L.F. (1979). Polysulfur nitride - a metallic, superconducting polymer. Chem Rev.

[95] Le T.-H., Kim Y., Yoon H. (2017). Electrical and electrochemical properties of conducting polymers. Polymers.

[96] Bredas J.L., Street G.B. (1985). Polarons, bipolarons, and solitons in conducting polymers. Acc. Chem. Res..

[97] Roth S., Bleier H. (1987). Solitons in polyacetylene. Adv. Phys..

[98] de Leeuw D.M., Simenon M.M.J., Brown A.R., Einerhand R.E.F. (1997). Stability of n-type doped conducting polymers and consequences for polymeric microelectronic devices. Synth. Met..

[99] Bajpai M., Srivastava R., Dhar R., Tiwari R.S. (2016). Review on optical and electrical properties of conducting polymers. Indian Journal of Materials Science.

[100] Nollau A., Pfeiffer M., Fritz T., Leo K. (2000). Controlled *n* -type doping of a molecular organic semiconductor: naphthalenetetracarboxylic dianhydride (NTCDA) doped with bis(ethylenedithio)-tetrathiafulvalene (BEDT-TTF). J. Appl. Phys..

[101] Zhang Y., Blom P.W.M. (2011). Electron and hole transport in poly(fluorene-benzothiadiazole). Appl. Phys. Lett..

[102] Zhang Y., de Boer B., Blom P.W.M. (2009). Controllable molecular doping and charge transport in solution‐processed polymer semiconducting layers. Adv. Funct. Mater..

[103] Anderson P.W. (1975). Model for the electronic structure of amorphous semiconductors. Phys. Rev. Lett..

[104] Mdluli S.B., Ramoroka M.E., Yussuf S.T., Modibane K.D., John-Denk V.S., Iwuoha E.I. (2022). π-Conjugated polymers and their application in organic and hybrid organic-silicon solar cells. Polymers.

[105] Heeger A.J., Wudl F. (1988).

[106] Sullivan E.M., Oh Y.J., Gerhardt R.A., Wang B., Kalaitzidou K. (2014). Understanding the effect of polymer crystallinity on the electrical conductivity of exfoliated graphite nanoplatelet/polylactic acid composite films. J. Polym. Res..

[107] Tsukamoto J. (1992). Recent advances in highly conductive polyacetylene. Adv. Phys..

[108] Chen X.B., Issi J.-P., Cassart M., Devaux J., Billaud D. (1994). Temperature dependence of the conductivity in conducting polymer composites. Polymer.

[109] Shaktawat V., Jain N., Saxena R., Saxena N.S., Sharma K., Sharma T.P. (2006). Temperature dependence of electrical conduction in pure and doped polypyrrole. Polym. Bull..

[110] Reicha F.M., Ahmed M.A., El‐Nimer M., El‐Sonbati A.Z., Diab M.A. (1989). Conducting polymers. III. Frequency and temperature dependence of the electrical conductivity of poly(bis‐2,6‐diaminopyridine sulphoxide). Acta Polym..

[111] Maddison D.S., Unsworth J., Roberts R.B. (1988). Electrical conductivity and thermoelectric power of polypyrrole with different doping levels. Synth. Met..

[112] Kuwabara M., Abe S. (1997).

[113] Stafström S., Fagerstrm J. (1994). Effects of interchain interactions on the localization of doping induced defects in quasi one-dimensional systems, Molecular Crystals and Liquid Crystals Science and Technology. Section A. Mol. Cryst. Liq. Cryst..

[114] Edwards P., Kuznetsov V., Slocombe D., Vijayaraghavan R. (2013). Comprehensive Inorganic Chemistry II.

[115] Schäfer-Sieber D., Roth S. (1989). Limitation of the conductivity of polyacetylene by conjugational defects. Synth. Met..

[116] Sheng P. (1980). Fluctuation-induced tunneling conduction in disordered materials. Phys. Rev. B.

[117] Burroughes J.H., Bradley D.D.C., Brown A.R., Marks R.N., Mackay K., Friend R.H., Burns P.L., Holmes A.B. (1990). Light-emitting diodes based on conjugated polymers. Nature.

[118] Geffroy B., le Roy P., Prat C. (2006). Organic light‐emitting diode (OLED) technology: materials, devices and display technologies. Polym. Int..

[119] Kulkarni A.P., Tonzola C.J., Babel A., Jenekhe S.A. (2004). Electron transport materials for organic light-emitting diodes. Chem. Mater..

[120] Pittaluga M. (2015). Eco-Efficient Materials for Mitigating Building Cooling Needs.

[121] Fink J.K. (2014). High Perform Polym.

[122] Kakaei K., Esrafili M.D., Ehsani A. (2019).

[123] Brooke R., Edberg J., Iandolo D., Berggren M., Crispin X., Engquist I. (2018). Controlling the electrochromic properties of conductive polymers using UV-light. J Mater Chem C Mater.

[124] Pagès H., Topart P., Lemordant D. (2001). Wide band electrochromic displays based on thin conducting polymer films. Electrochim. Acta.

[125] Barnes A., Despotakis A., Wong T.C.P., Anderson A.P., Chambers B., V Wright P. (1998). Towards a ′smart window’ for microwave applications. Smart Mater. Struct..

[126] Hyodo K. (1994). Electrochromism of conducting polymers. Electrochim. Acta.

[127] Buitrago E., Novello A.M., Meyer T. (2020). Third‐generation solar cells: toxicity and risk of exposure. Helv. Chim. Acta.

[128] Hoppe H., Sariciftci N.S. (2004). Organic solar cells: an overview. J. Mater. Res..

[129] Ghosh A.K., Feng T. (1978). Merocyanine organic solar cells. J. Appl. Phys..

[130] Morel D.L., Ghosh A.K., Feng T., Stogryn E.L., Purwin P.E., Shaw R.F., Fishman C. (1978). High-efficiency organic solar cells. Appl. Phys. Lett..

[131] Tang C.W. (1986). Two-layer organic photovoltaic cell. Appl. Phys. Lett..

[132] Markov D.E., Amsterdam E., Blom P.W.M., Sieval A.B., Hummelen J.C. (2005). Accurate measurement of the exciton diffusion length in a conjugated polymer using a heterostructure with a side-chain cross-linked fullerene layer. J. Phys. Chem. A.

[133] Stübinger T., Brütting W. (2001). Exciton diffusion and optical interference in organic donor–acceptor photovoltaic cells. J. Appl. Phys..

[134] Haugeneder A., Neges M., Kallinger C., Spirkl W., Lemmer U., Feldmann J., Scherf U., Harth E., Gügel A., Müllen K. (1999). Exciton diffusion and dissociation in conjugated polymer/fullerene blends and heterostructures. Phys. Rev. B.

[135] M. Theander, A. Yartsev, D. Zigmantas, V. Sundströ, W. Mammo, M.R. Andersson, O. Inganä, Photoluminescence Quenching at a polythiopheneÕC 60 Heterojunction, n.d.

[136] Halls J.J.M., Pichler K., Friend R.H., Moratti S.C., Holmes A.B. (1996). Exciton diffusion and dissociation in a poly(*p* -phenylenevinylene)/C60 heterojunction photovoltaic cell. Appl. Phys. Lett..

[137] Dennler G., Scharber M.C., Brabec C.J. (2009). Polymer‐fullerene bulk‐heterojunction solar cells. Adv. Mater..

[138] Yu G., Gao J., Hummelen J.C., Wudl F., Heeger A.J. (1979). Polymer photovoltaic cells: enhanced efficiencies via a network of internal donor-acceptor heterojunctions. Science.

[139] Zhou H., Yang L., You W. (2012). Rational design of high performance conjugated polymers for organic solar cells. Macromolecules.

[140] Cheng Y.-J., Yang S.-H., Hsu C.-S. (2009). Synthesis of conjugated polymers for organic solar cell applications. Chem Rev.

[141] Xu T., Yu L. (2014). How to design low bandgap polymers for highly efficient organic solar cells. Mater. Today.

[142] Yan C., Barlow S., Wang Z., Yan H., Jen A.K.-Y., Marder S.R., Zhan X. (2018). Non-fullerene acceptors for organic solar cells. Nat. Rev. Mater..

[143] Hong L., Yao H., Cui Y., Bi P., Zhang T., Cheng Y., Zu Y., Qin J., Yu R., Ge Z., Hou J. (2021). 18.5% efficiency organic solar cells with a hybrid planar/bulk heterojunction. Adv. Mater..

[144] Coakley K.M., McGehee M.D. (2004). Conjugated polymer photovoltaic cells. Chem. Mater..

[145] Fu J., Fong P.W.K., Liu H., Huang C.-S., Lu X., Lu S., Abdelsamie M., Kodalle T., Sutter-Fella C.M., Yang Y., Li G. (2023). 19.31% binary organic solar cell and low non-radiative recombination enabled by non-monotonic intermediate state transition. Nat. Commun..

[146] Attia N.F., Lee S.M., Kim H.J., Geckeler K.E. (2014). Nanoporous polypyrrole: preparation and hydrogen storage properties. Int. J. Energy Res..

[147] Mahato N., Jang H., Dhyani A., Cho S. (2020). Recent progress in conducting polymers for hydrogen storage and fuel cell applications. Polymers.

[148] Kamarudin S.K., Achmad F., Daud W.R.W. (2009). Overview on the application of direct methanol fuel cell (DMFC) for portable electronic devices. Int. J. Hydrogen Energy.

[149] Wei Z.D., Chan S.H. (2004). Electrochemical deposition of PtRu on an uncatalyzed carbon electrode for methanol electrooxidation. J. Electroanal. Chem..

[150] Madaswamy S.L., Alothman A.A., mana Al-Anazy M., Ifseisi A.A., Alqahtani K.N., Natarajan S.K., Angaiah S., Ragupathy D. (2021). Polyaniline-based nanocomposites for direct methanol fuel cells (DMFCs) - a Recent Review. J. Ind. Eng. Chem..

[151] Kim J., Kim J.H., Ariga K. (2017). Redox-active polymers for energy storage nanoarchitectonics. Joule.

[152] Hager M.D., Esser B., Feng X., Schuhmann W., Theato P., Schubert U.S. (2020). Polymer‐based batteries—flexible and thin energy storage systems. Adv. Mater..

[153] Chen J., Cheng F. (2009). Combination of lightweight elements and nanostructured materials for batteries. Acc. Chem. Res..

[154] Kang K., Meng Y.S., Bréger J., Grey C.P., Ceder G. (2006). Electrodes with high power and high capacity for rechargeable lithium batteries. Science.

[155] Whittingham M.S. (2004). Lithium batteries and cathode materials. Chem Rev.

[156] Armand M., Tarascon J.-M. (2008). Building better batteries. Nature.

[157] Winter M., Brodd R.J. (2004). What are batteries, fuel cells, and supercapacitors?. Chem Rev.

[158] Sengodu P., Deshmukh A.D. (2015). Conducting polymers and their inorganic composites for advanced Li-ion batteries: a review. RSC Adv..

[159] Singh N., Kumar A., Riaz U. (2022). Conducting polymer‐based micro‐ and nano‐batteries for biomedical applications: a short review. ChemistrySelect.

[160] Meng Q., Cai K., Chen Y., Chen L. (2017). Research progress on conducting polymer based supercapacitor electrode materials. Nano Energy.

[161] Bryan A.M., Santino L.M., Lu Y., Acharya S., D'Arcy J.M. (2016). Conducting polymers for pseudocapacitive energy storage. Chem. Mater..

[162] Ahmed S., Ahmed A., Basha D.B., Hussain S., Uddin I., Gondal M.A. (2023). Critical review on recent developments in conducting polymer nanocomposites for supercapacitors. Synth. Met..

[163] Han Y., Dai L. (2019). Conducting polymers for flexible supercapacitors. Macromol. Chem. Phys..

[164] Mastragostino M., Arbizzani C., Soavi F. (2001). Polymer-based supercapacitors. J. Power Sources.

[165] Ryu K.S., Lee Y.-G., Hong Y.-S., Park Y.J., Wu X., Kim K.M., Kang M.G., Park N.-G., Chang S.H. (2004). Poly(ethylenedioxythiophene) (PEDOT) as polymer electrode in redox supercapacitor. Electrochim. Acta.

[166] Snook G.A., Kao P., Best A.S. (2011). Conducting-polymer-based supercapacitor devices and electrodes. J. Power Sources.

[167] (2019). A review on conducting polymers-based composites for energy storage application. Journal of Chemical Reviews.

[168] Freitas B., Nunes W.G., Soares D.M., Rufino F.C., Moreira C.M., Da Silva L.M., Zanin H. (2021). Robust, flexible, freestanding and high surface area activated carbon and multi-walled carbon nanotubes composite material with outstanding electrode properties for aqueous-based supercapacitors. Mater Adv.

[169] Gul H., Shah A.-H.A., Krewer U., Bilal S. (2020). Study on direct synthesis of energy efficient multifunctional polyaniline–graphene oxide nanocomposite and its application in aqueous symmetric supercapacitor devices. Nanomaterials.

[170] Prasankumar T., Wiston B.R., Gautam C.R., Ilangovan R., Jose S.P. (2018). Synthesis and enhanced electrochemical performance of PANI/Fe3O4 nanocomposite as supercapacitor electrode. J. Alloys Compd..

[171] Kalambate P.K., Dar R.A., Karna S.P., Srivastava A.K. (2015). High performance supercapacitor based on graphene-silver nanoparticles-polypyrrole nanocomposite coated on glassy carbon electrode. J. Power Sources.

[172] Zhang C., Xu S., Cai D., Cao J., Wang L., Han W. (2020). Planar supercapacitor with high areal capacitance based on Ti3C2/Polypyrrole composite film. Electrochim. Acta.

[173] Chen X., Zhu X., Xiao Y., Yang X. (2015). PEDOT/g-C3N4 binary electrode material for supercapacitors. J. Electroanal. Chem..

[174] Jabeen N., Xia Q., Yang M., Xia H. (2016). Unique core–shell nanorod arrays with polyaniline deposited into mesoporous NiCo _2_ O _4_ support for high-performance supercapacitor electrodes. ACS Appl. Mater. Interfaces.

[175] Sk M.M., Yue C.Y., Jena R.K. (2015). Non-covalent interactions and supercapacitance of pseudo-capacitive composite electrode materials (MWCNTCOOH/MnO2/PANI). Synth. Met..

[176] Balboni R.D.C., Maron G.K., Masteghin M.G., Tas M.O., Rodrigues L.S., Gehrke V., Alano J.H., Andreazza R., Carreño N.L.V., Silva S.R.P. (2022). An easy to assemble PDMS/CNTs/PANI flexible supercapacitor with high energy-to-power density. Nanoscale.

[177] Li J., Lu W., Yan Y., Chou T.-W. (2017). High performance solid-state flexible supercapacitor based on Fe _3_ O _4_/carbon nanotube/polyaniline ternary films. J Mater Chem A Mater.

[178] AlSalhi M.S., Alam J., Dass L.A., Raja M. (2011). Recent advances in conjugated polymers for light emitting devices. Int. J. Mol. Sci..

[179] Gupta R.C., Milatovic D., Lall R., Srivastava A. (2018). Veterinary Toxicology.

[180] AlSalhi M.S., Alam J., Dass L.A., Raja M. (2011). Recent advances in conjugated polymers for light emitting devices. Int. J. Mol. Sci..

[181] Wong W.W.H., Banal J.L. (2015). Encyclopedia of Polymeric Nanomaterials.

[182] Chen S.-A., Chuang K.-R., Chao C.-I., Lee H.-T. (1996). White-light emission from electroluminescence diode with polyaniline as the emitting layer. Synth. Met..

[183] Yang Y., Westerweele E., Zhang C., Smith P., Heeger A.J. (1995). Enhanced performance of polymer light-emitting diodes using high-surface area polyaniline network electrodes. J. Appl. Phys..

[184] Kaminorz Y., Smela E., Johansson T., Brehmer L., Andersson M.R., Inganäs O. (2000). Characteristics of polythiophene surface light emitting diodes. Synth. Met..

[185] Perepichka I.F., Perepichka D.F., Meng H., Wudl F. (2005). Light‐emitting polythiophenes. Adv. Mater..

[186] Cazeca M.J., Chittibabu K.G., Kim J., Kumar J., Jain A., Kim W., Tripathy S.K. (1998). Enhanced performance of polythiophene derivative based light emitting diodes by addition of europium and ruthenium complexes. Synth. Met..

[187] Kulkarni A.P., Jenekhe S.A. (2003). Blue light-emitting diodes with good spectral stability based on blends of poly(9,9-dioctylfluorene): interplay between morphology, photophysics, and device performance. Macromolecules.

[188] Bernius M., Inbasekaran M., Woo E., Wu W., Wujkowski L. (2000). Light-emitting diodes based on fluorene polymers. Thin Solid Films.

[189] Lindgren L.J., Zhang F., Admassie S., Wang X., Andersson M.R., Inganäs O. (2007). Blue light-emitting diodes based on novel polyfluorene copolymers. J. Lumin..

[190] Petsagkourakis I., Kim N., Tybrandt K., Zozoulenko I., Crispin X. (2019). Poly(3,4‐ethylenedioxythiophene): chemical synthesis, transport properties, and thermoelectric devices. Adv Electron Mater.

[191] Sharma S., Sudhakara P., Omran A.A.B., Singh J., Ilyas R.A. (2021). Recent trends and developments in conducting polymer nanocomposites for multifunctional applications. Polymers.

[192] Argun A.A., Aubert P.-H., Thompson B.C., Schwendeman I., Gaupp C.L., Hwang J., Pinto N.J., Tanner D.B., MacDiarmid A.G., Reynolds J.R. (2004). Multicolored electrochromism in polymers: structures and devices. Chem. Mater..

[193] Zhang S., Ren J., Chen S., Luo Y., Bai X., Ye L., Yang F., Cao Y. (2020). Large area electrochromic displays with ultrafast response speed and high contrast using solution-processable and patternable honeycomb-like polyaniline nanostructures. J. Electroanal. Chem..

[194] Somani P., Mandale A.B., Radhakrishnan S. (2000). Study and development of conducting polymer-based electrochromic display devices. Acta Mater..

[195] Park C., Kim J.M., Kim Y., Bae S., Do M., Im S., Yoo S., Kim J.H. (2021). High-coloration efficiency and low-power consumption electrochromic film based on multifunctional conducting polymer for large scale smart windows. ACS Appl. Electron. Mater..

[196] Howard E.L., Österholm A.M., Shen D.E., Panchumarti L.P., Pinheiro C., Reynolds J.R. (2021). Cost-effective, flexible, and colorful dynamic displays: removing underlying conducting layers from polymer-based electrochromic devices. ACS Appl. Mater. Interfaces.

[197] Deshpande P.P., Jadhav N.G., Gelling V.J., Sazou D. (2014). Conducting polymers for corrosion protection: a review. J Coat Technol Res.

[198] Xu H., Zhang Y. (2019). A review on conducting polymers and nanopolymer composite coatings for steel corrosion protection. Coatings.

[199] Grundmeier G., Schmidt W., Stratmann M. (2000). Corrosion protection by organic coatings: electrochemical mechanism and novel methods of investigation. Electrochim. Acta.

[200] Grgur B.N., Gvozdenović M.M., Mišković-Stanković V.B., Kačarević-Popović Z. (2006). Corrosion behavior and thermal stability of electrodeposited PANI/epoxy coating system on mild steel in sodium chloride solution. Prog Org Coat.

[201] Fihri A., Bovero E., Al-Shahrani A., Al-Ghamdi A., Alabedi G. (2017). Recent progress in superhydrophobic coatings used for steel protection: a review. Colloids Surf. A Physicochem. Eng. Asp..

[202] Kadri Z., Mechnou I., Zyade S. (2021). Migration of bisphenol A from epoxy coating to foodstuffs. Mater Today Proc.

[203] Vermeirssen E.L.M., Dietschweiler C., Werner I., Burkhardt M. (2017). Corrosion protection products as a source of bisphenol A and toxicity to the aquatic environment. Water Res..

[204] Deshpande P.P., Jadhav N.G., Gelling V.J., Sazou D. (2014). Conducting polymers for corrosion protection: a review. J Coat Technol Res.

[205] Tallman D.E., Spinks G., Dominis A., Wallace G.G. (2002). Electroactive conducting polymers for corrosion control. J. Solid State Electrochem..

[206] Ohtsuka T. (2012). Corrosion protection of steels by conducting polymer coating. International Journal of Corrosion.

[207] Herrasti P., Recio F.J., Ocón P., Fatás E. (2005). Effect of the polymer layers and bilayers on the corrosion behaviour of mild steel: comparison with polymers containing Zn microparticles. Prog Org Coat.

[208] Kinlen P.J., Menon V., Ding Y. (1999). A mechanistic investigation of polyaniline corrosion protection using the scanning reference electrode technique. J. Electrochem. Soc..

[209] DeBerry D.W. (1985). Modification of the electrochemical and corrosion behavior of stainless steels with an electroactive coating. J. Electrochem. Soc..

[210] Wessling B. (1994). Passivation of metals by coating with polyaniline: corrosion potential shift and morphological changes. Adv. Mater..

[211] Gašparac R., Martin C.R. (2001). Investigations of the mechanism of corrosion inhibition by polyaniline. Polyaniline-coated stainless steel in sulfuric acid solution. J. Electrochem. Soc..

[212] Herrasti P., Ocón P., Ibáñez A., Fatás E. (2003). Electroactive polymer films for stainless steel corrosion protection. J. Appl. Electrochem..

[213] Yahalom J. (2001). Encyclopedia of Materials: Science and Technology.

[214] Ohtsuka T. (2012). Corrosion protection of steels by conducting polymer coating. International Journal of Corrosion.

[215] Shabani-Nooshabadi M., Ghoreishi S.M., Behpour M. (2009). Electropolymerized polyaniline coatings on aluminum alloy 3004 and their corrosion protection performance. Electrochim. Acta.

[216] Mobin M., Ansar F. (2022). Polythiophene (PTh)–TiO _2_ –reduced graphene oxide (rGO) nanocomposite coating: synthesis, characterization, and corrosion protection performance on low-carbon steel in 3.5 wt % NaCl solution. ACS Omega.

[217] Muthusamy P., Konda Kannan S.K. (2021). High efficient corrosion inhibitor of <scp>water‐soluble</scp> polypyrrole–sulfonated melamine formaldehyde nanocomposites for <scp>316 L</scp> stainless steel. J. Appl. Polym. Sci..

[218] Olad A., Barati M., Behboudi S. (2012). Preparation of PANI/epoxy/Zn nanocomposite using Zn nanoparticles and epoxy resin as additives and investigation of its corrosion protection behavior on iron. Prog Org Coat.

[219] Sathiyanarayanan S., Syed Azim S., Venkatachari G. (2007). Preparation of polyaniline–TiO2 composite and its comparative corrosion protection performance with polyaniline. Synth. Met..

[220] Mobin M., Ansar F. (2022). Polythiophene (PTh)–TiO _2_ –reduced graphene oxide (rGO) nanocomposite coating: synthesis, characterization, and corrosion protection performance on low-carbon steel in 3.5 wt % NaCl solution. ACS Omega.

[221] Shabani-Nooshabadi M., Ghoreishi S.M., Behpour M. (2009). Electropolymerized polyaniline coatings on aluminum alloy 3004 and their corrosion protection performance. Electrochim. Acta.

[222] Bandeira R.M., van Drunen J., Tremiliosi-Filho G., dos Santos J.R., de Matos J.M.E. (2017). Polyaniline/polyvinyl chloride blended coatings for the corrosion protection of carbon steel. Prog Org Coat.

[223] Sakhri A., Perrin F.X., Benaboura A., Aragon E., Lamouri S. (2011). Corrosion protection of steel by sulfo-doped polyaniline-pigmented coating. Prog Org Coat.

[224] Parwaz Khan A.A., Singh P., Raizada P., Khan A., Asiri A.M., Alotaibi M.M. (2023). Photo-Fenton assisted AgCl and P-doped g-C3N4 Z-scheme photocatalyst coupled with Fe3O4/H2O2 system for 2, 4-dimethylphenol degradation. Chemosphere.

[225] Malhotra M., Poonia K., Singh P., Khan A.A.P., Thakur P., Van Le Q., Helmy E.T., Ahamad T., Nguyen V.-H., Thakur S., Raizada P. (2024). An overview of improving photocatalytic activity of MnO2 via the Z-scheme approach for environmental and energy applications. J. Taiwan Inst. Chem. Eng..

[226] Dhull P., Sudhaik A., Sharma V., Raizada P., Hasija V., Gupta N., Ahamad T., Nguyen V.-H., Kim A., Shokouhimehr M., Kim S.Y., Van Le Q., Singh P. (2023). An overview on InVO4-based photocatalysts: electronic properties, synthesis, enhancement strategies, and photocatalytic applications. Mol. Catal..

[227] Tran H.-T., Bolan N.S., Lin C., Binh Q.A., Nguyen M.-K., Luu T.A., Le V.-G., Pham C.Q., Hoang H.-G., Vo D.-V.N. (2023). Succession of biochar addition for soil amendment and contaminants remediation during co-composting: a state of art review. J Environ Manage.

[228] Jiang M., Ye K., Deng J., Lin J., Ye W., Zhao S., Van der Bruggen B. (2018). Conventional ultrafiltration as effective strategy for dye/salt fractionation in textile wastewater treatment. Environ. Sci. Technol..

[229] Lellis B., Fávaro-Polonio C.Z., Pamphile J.A., Polonio J.C. (2019). Effects of textile dyes on health and the environment and bioremediation potential of living organisms. Biotechnology Research and Innovation.

[230] Aquino J.M., Rocha-Filho R.C., Ruotolo L.A.M., Bocchi N., Biaggio S.R. (2014). Electrochemical degradation of a real textile wastewater using β-PbO2 and DSA® anodes. Chem. Eng. J..

[231] Khatri J., Nidheesh P.V., Anantha Singh T.S., Suresh Kumar M. (2018). Advanced oxidation processes based on zero-valent aluminium for treating textile wastewater. Chem. Eng. J..

[232] Kenawy E.-R., Ghfar A.A., Wabaidur S.M., Khan M.A., Siddiqui M.R., Alothman Z.A., Alqadami A.A., Hamid M. (2018). Cetyltrimethylammonium bromide intercalated and branched polyhydroxystyrene functionalized montmorillonite clay to sequester cationic dyes. J Environ Manage.

[233] Khan M.A., Wabaidur S.M., Siddiqui M.R., Alqadami A.A., Khan A.H. (2020). Silico-manganese fumes waste encapsulated cryogenic alginate beads for aqueous environment de-colorization. J. Clean. Prod..

[234] Ali I., Alharbi O.M.L., Alothman Z.A., Al-Mohaimeed A.M., Alwarthan A. (2019). Modeling of fenuron pesticide adsorption on CNTs for mechanistic insight and removal in water. Environ. Res..

[235] Al-Nuaim M.A., Alwasiti A.A., Shnain Z.Y. (2023). The photocatalytic process in the treatment of polluted water. Chem. Pap..

[236] Saravanan R., Sacari E., Gracia F., Khan M.M., Mosquera E., Gupta V.K. (2016). Conducting PANI stimulated ZnO system for visible light photocatalytic degradation of coloured dyes. J. Mol. Liq..

[237] Ramalingam G., Perumal N., Priya A.K., Rajendran S. (2022). A review of graphene-based semiconductors for photocatalytic degradation of pollutants in wastewater. Chemosphere.

[238] Wang X., Zhang J., Zhang K., Zou W., Chen S. (2016). Facile fabrication of reduced graphene oxide/CuI/PANI nanocomposites with enhanced visible-light photocatalytic activity. RSC Adv..

[239] Abid P. Sehrawat, Islam S.S., Mishra P., Ahmad S. (2018). Reduced graphene oxide (rGO) based wideband optical sensor and the role of Temperature, Defect States and Quantum Efficiency. Sci. Rep..

[240] Al Kausor M., Chakrabortty D. (2021). Graphene oxide based semiconductor photocatalysts for degradation of organic dye in waste water: a review on fabrication, performance enhancement and challenges. Inorg. Chem. Commun..

[241] Soni V., Khosla A., Singh P., Nguyen V.-H., Van Le Q., Selvasembian R., Hussain C.M., Thakur S., Raizada P. (2022). Current perspective in metal oxide based photocatalysts for virus disinfection: a review. J Environ Manage.

[242] Mitra M., Ahamed SkT., Ghosh A., Mondal A., Kargupta K., Ganguly S., Banerjee D. (2019). Polyaniline/reduced graphene oxide composite-enhanced visible-light-driven photocatalytic activity for the degradation of organic dyes. ACS Omega.

[243] Sambaza S.S., Maity A., Pillay K. (2020). Polyaniline-coated TiO _2_ nanorods for photocatalytic degradation of bisphenol A in water. ACS Omega.

[244] Vidya J., Balamurugan P. (2019). Photocatalytic degradation of methylene blue using PANi—NiO nanocomposite under visible light irradiation. Mater. Res. Express.

[245] Wang F., Min S., Han Y., Feng L. (2010). Visible-light-induced photocatalytic degradation of methylene blue with polyaniline-sensitized composite photocatalysts. Superlattices Microstruct.

[246] Sharma S., Sharma G., Kumar A., Naushad Mu, Mola G.T., Kumar A., Al-Misned F.A., El-Serehy H.A., Stadler F.J. (2020). Visibly active FeO/ZnO@PANI magnetic nano-photocatalyst for the degradation of 3-aminophenol. Top. Catal..

[247] Deng F., Li Y., Luo X., Yang L., Tu X. (2012). Preparation of conductive polypyrrole/TiO2 nanocomposite via surface molecular imprinting technique and its photocatalytic activity under simulated solar light irradiation. Colloids Surf. A Physicochem. Eng. Asp..

[248] Mittal H., Khanuja M. (2021). Hydrothermal in-situ synthesis of MoSe2-polypyrrole nanocomposite for efficient photocatalytic degradation of dyes under dark and visible light irradiation. Sep. Purif. Technol..

[249] Zhang L., Jamal R., Zhao Q., Wang M., Abdiryim T. (2015). Preparation of PEDOT/GO, PEDOT/MnO2, and PEDOT/GO/MnO2 nanocomposites and their application in catalytic degradation of methylene blue. Nanoscale Res. Lett..

[250] Zia J., Aazam E.S., Riaz U. (2020). Highly efficient visible light driven photocatalytic activity of MnO2 and Polythiophene/MnO2 nanohybrids against mixed organic pollutants. J. Mol. Struct..

[251] Xu S., Jiang L., Yang H., Song Y., Dan Y. (2011). Structure and photocatalytic activity of polythiophene/TiO2 composite particles prepared by photoinduced polymerization. Chin. J. Catal..

[252] Alothman Z. (2012). A review: fundamental aspects of silicate mesoporous materials. Materials.

[253] Jadoun S., Fuentes J.P., Urbano B.F., Yáñez J. (2023). A review on adsorption of heavy metals from wastewater using conducting polymer-based materials. J. Environ. Chem. Eng..

[254] Jadoun S., Fuentes J.P., Urbano B.F., Yáñez J. (2023). A review on adsorption of heavy metals from wastewater using conducting polymer-based materials. J. Environ. Chem. Eng..

[255] Khan M.I., Almesfer M.K., Elkhaleefa A., Shigidi I., Shamim M.Z., Ali I.H., Rehan M. (2021). Conductive polymers and their nanocomposites as adsorbents in environmental applications. Polymers.

[256] Zhou T., Liang Q., Zhou X., Luo H., Chen W. (2021). Enhanced removal of toxic hexavalent chromium from aqueous solution by magnetic Zr-MOF@polypyrrole: performance and mechanism. Environ. Sci. Pollut. Control Ser..

[257] Samadi A., Xie M., Li J., Shon H., Zheng C., Zhao S. (2021). Polyaniline-based adsorbents for aqueous pollutants removal: a review. Chem. Eng. J..

[258] Wang N., Li J., Lv W., Feng J., Yan W. (2015). Synthesis of polyaniline/TiO _2_ composite with excellent adsorption performance on acid red G. RSC Adv..

[259] Mosavi S.S., Zare E.N., Behniafar H., Tajbakhsh M. (2023). Removal of amoxicillin antibiotic from polluted water by a magnetic bionanocomposite based on carboxymethyl tragacanth gum-grafted-polyaniline. Water (Basel).

[260] Senguttuvan S., Janaki V., Senthilkumar P., Kamala-Kannan S. (2022). Polypyrrole/zeolite composite – a nanoadsorbent for reactive dyes removal from synthetic solution. Chemosphere.

[261] Haghgir A., Hosseini S.H., Tanzifi M., Tavakkoli Yaraki M., Bayati B., Saemian T., Koohi M. (2022). Synthesis of polythiophene/zeolite/iron nanocomposite for adsorptive remediation of azo dye: optimized by Taguchi method. Chem. Eng. Res. Des..

[262] Kharazi P., Rahimi R., Rabbani M. (2019). Copper ferrite-polyaniline nanocomposite: structural, thermal, magnetic and dye adsorption properties. Solid State Sci..

[263] Chen J., Dong R., Chen S., Tang D., Lou X., Ye C., Qiu T., Yan W. (2022). Selective adsorption towards heavy metal ions on the green synthesized polythiophene/MnO2 with a synergetic effect. J. Clean. Prod..

[264] Zhang H., Ding X., Wang S., Huang Y., Zeng X.-F., Maganti S., Jiang Q., Huang M., Guo Z., Cao D. (2022). Heavy metal removal from wastewater by a polypyrrole-derived N-doped carbon nanotube decorated with fish scale-like molybdenum disulfide nanosheets. Engineered Science.

[265] Anuma S., Mishra P., Bhat B.R. (2021). Polypyrrole functionalized Cobalt oxide Graphene (COPYGO) nanocomposite for the efficient removal of dyes and heavy metal pollutants from aqueous effluents. J. Hazard Mater..

[266] Du L., Gao P., Meng Y., Liu Y., Le S., Yu C. (2020). Highly efficient removal of Cr(VI) from aqueous solutions by polypyrrole/monodisperse latex spheres. ACS Omega.

[267] Ravichandran R., Sundarrajan S., Venugopal J.R., Mukherjee S., Ramakrishna S. (2010). Applications of conducting polymers and their issues in biomedical engineering. J R Soc Interface.

[268] Majeed K. (2012). New Polymers for Special Applications.

[269] Das T.K., Prusty S. (2012). Review on conducting polymers and their applications. Polym. Plast. Technol. Eng..

[270] Cirera B., Sánchez-Grande A., de la Torre B., Santos J., Edalatmanesh S., Rodríguez-Sánchez E., Lauwaet K., Mallada B., Zbořil R., Miranda R., Gröning O., Jelínek P., Martín N., Ecija D. (2020). Tailoring topological order and π-conjugation to engineer quasi-metallic polymers. Nat. Nanotechnol..

